# Circular RNAs in cancer: Its biogenesis, functions, relationships with cancer progression, applications in immunotherapy and biomarker potentials

**DOI:** 10.1007/s00262-025-04176-z

**Published:** 2026-03-31

**Authors:** Yusen Gao, Lingling Xu, Ruihua Sun, Luping Gao, Peiyu Yan, Xinrong Yang, Guoliang Wang, Yanfang Xian, Jiewen Zhang, Di Zhu

**Affiliations:** 1 School of Pharmacy, Fudan University, Shanghai, China; 2 Zhejiang MegaLife Health Technology Group Co., Ltd, Hangzhou, China; 3 Department of Neurology, Henan Provincial People’s Hospital, Zhengzhou University People’s Hospital, Zhengzhou, 450003 Henan China; 4 Guangxi Key Laboratory of Tumor Immunology and Microenvironmental Regulation, Guilin Medical University, Guilin, 541004 China; 5 Faculty of Chinese Medicine, National Key Laboratory of Mechanism and Quality of Chinese Medicines, Macau University of Science and Technology, Macau, 999078 China; 6 Zhuhai MUST Science and Technology Research Institute, Zhuhai, 519031 China; 7 Department of Hepatobiliary Surgery & Transplantation, Liver Cancer Institute, Zhongshan Hospital, Fudan University, and Key Laboratory of Carcinogenesis and Cancer Invasion, Ministry of Education, Shanghai, 200032 China; 8 UniTTEC Co., Ltd, Hangzhou, China; 9 School of Chinese Medicine, Faculty of Medicine, The Chinese University of Hong Kong, Shatin, N.T., Hong Kong SAR, China; 10 Department of Pharmacology, School of Basic Medical Sciences, Fudan University, Shanghai, 200433 China; 11 Key Laboratory of Tumor Immunology and Microenvironmental Regulation, and Department of Oncology, Second Affiliated Hospital of Guilin Medical University, Guangxi, 541001 China

**Keywords:** Biomarkers, CircRNAs, Cancer immunotherapy, CAR-T therapy, Immune checkpoint blockade, Tumor microenvironment

## Abstract

Circular RNAs (circRNAs) are covalently closed, single-stranded RNAs generated via backsplicing. They are highly stable and evolutionarily conserved, making them promising candidates for cancer therapy and diagnosis. CircRNAs regulate cancer progression by modulating genome instability, angiogenesis, metastasis, stemness, and chemoresistance. They do so through mechanisms including microRNA (miRNA) sponging, protein interaction, translational templating, and transcription/translation regulation. CircRNAs play a critical role in cancer immunotherapy. They modulate immune checkpoint blockade (ICB) responses and cytokine secretion to reshape the tumor immune microenvironment (TME). CircRNAs also serve as stable platforms for neoantigen-based cancer vaccines and improve in vivo chimeric antigen receptor T cell (CAR-T) therapy by replacing unstable linear mRNA. Additionally, circRNAs are potential noninvasive biomarkers due to their abundance in body fluids and differential tumor-normal expression. Despite challenges such as unclear regulatory networks, off-target effects, and inefficient delivery, this review systematically summarizes the biogenesis of circRNAs, their functional mechanisms, their roles in cancer progression, and their applications in cancer immunotherapy. The review also highlights their utility as biomarkers and future translational directions, providing a focused overview of their potential to advance cancer immunotherapy.

**Supplementary Information:**

The online version supplementary material available at 10.1007/s00262-025-04176-z.

## Introduction

**Fig. 1:**
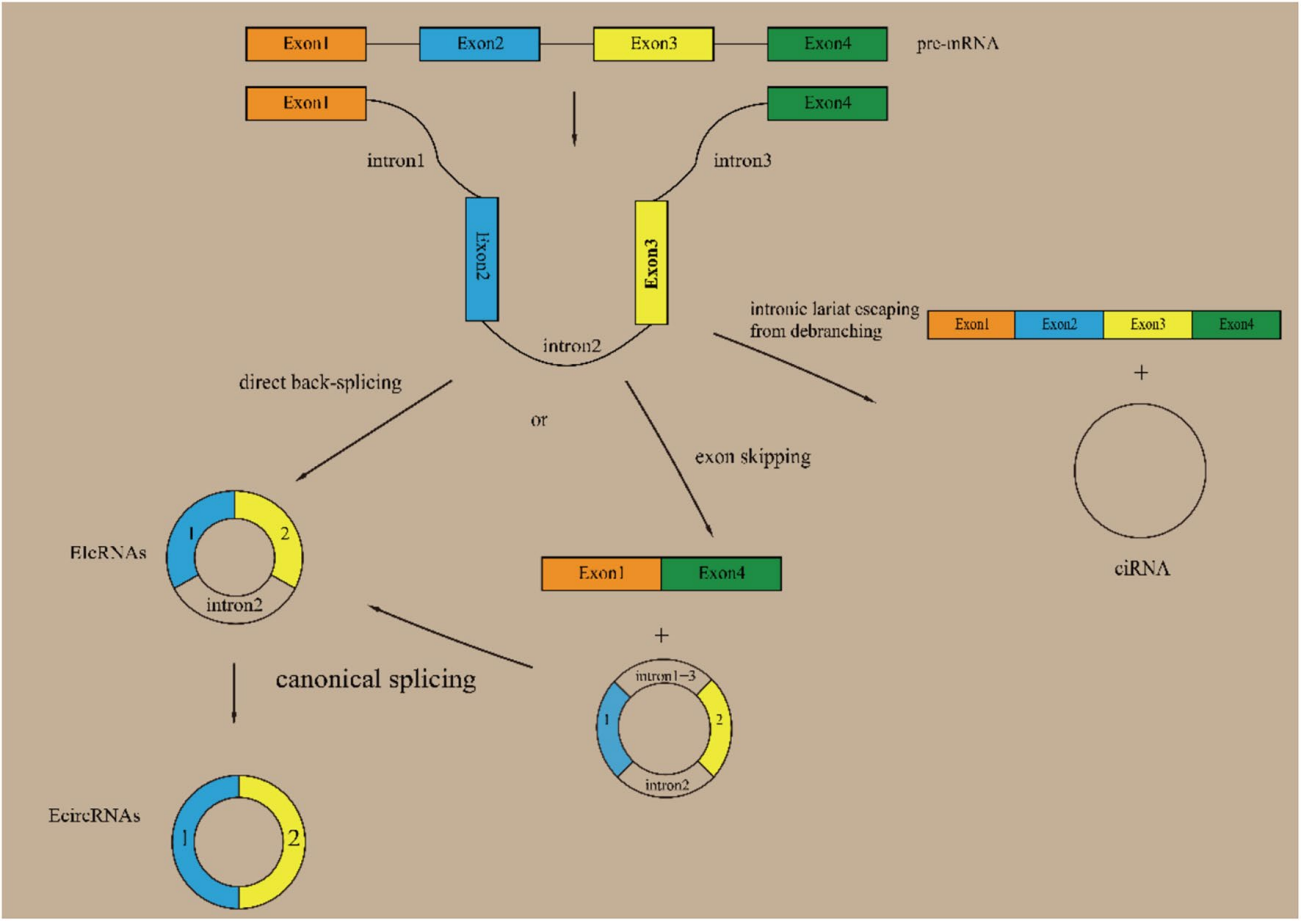
Biogenesis and Classification of Circular RNAs (circRNAs) Circular RNAs are derived from precursor messenger RNAs (pre-mRNAs) through back-splicing, a process that is relatively inefficient compared to the more prevalent canonical splicing. Two models have been proposed to explain the formation of circRNAs. The first model (on the left) is called “direct back-splicing.” This process begins with the back-splicing of Exon2 and Exon3. This results in the formation of an exon–intron circRNA (EIcRNA). This EIcRNA can undergo additional canonical splicing to form an exon circRNA (EcircRNA). The second model (on the right) presents two distinct approaches. "Exon skipping" is a splicing process that retains introns 1 and 3. This process is distinct from “direct back-splicing.” Consequently, “exon skipping” requires an additional canonical splicing step to generate a comparable EIcRNA. It has been observed that the intron 1 + 3 sequence can be retained as a new circRNA. “Intronic lariat escaping from debranching” refers to the process by which the intronic lariat precursor escapes the debranching step of canonical linear splicing, resulting in the formation of intronic circular RNA (ciRNA)

In vivo, circular RNAs (circRNAs) are generated by the spliceosome via back-splicing [1]. Their structure comprises a loop in which the 3’ and 5’ ends are covalently linked. The absence of 3’ and 5’ ends makes circRNAs resistant to exonuclease-mediated degradation, which gives them greater stability than linear mRNA [2]. CircRNAs are derived from pre-mRNAs transcribed by RNA polymerase II (Pol II); therefore, both canonical splice signals and spliceosomal machinery are required for back-splicing [3]. There are two possible models for back-splicing: “direct back-splicing” and “lariat intermediate.” The “lariat intermediate” model has two subtypes: “exon skipping” and “intronic lariat escaping from debranching” [4, 5]. According to their formation models and components, circRNAs can be classified into three categories: exon circRNAs (EcircRNAs), which are composed of exons; intron circRNAs (ciRNAs), which are composed of introns; and exon–intron circRNAs (EIciRNAs), which are composed of both exons and introns [2, 6, 7]. As illustrated in Fig. 1, the biogenesis and classification of circRNAs is a complex process.

A multitude of immunotherapeutic strategies for tumors are currently being actively explored and investigated. These include immune checkpoint inhibitors (ICIs), cytokine-based cancer immunotherapy, chimeric antigen receptor T cell therapy, and cancer vaccines. Each method has its own advantages and has generally demonstrated high efficacy. However, cancer cell proliferation may be resistant to these methods due to various factors, including a lack of antigen presentation, changes in crucial immune signaling pathways, immune cell depletion, and alternate immune checkpoint pathways [8, 9]. Research findings on circular RNAs (circRNAs) suggest a complex relationship with tumor development and a strong alignment with the principles of immunotherapy. Substantial research has revealed that many genes are transcribed into circular RNAs in human and mouse models, suggesting circRNAs are abundant in various organs and tissues [10, 11]. Moreover, circRNA dysregulation has been identified as a crucial factor in the spatial and temporal regulation of cancer progression [7]. For instance, circREEP3 is upregulated in colorectal cancer (CRC) tissues. circREEP3 knockout has been demonstrated to suppress CRC tumorigenesis and metastasis and impair the stem cell-like phenotype [12]. On the other hand, circHEATRF5B was expressed at low levels in glioblastoma multiforme (GBM). This circRNA has been shown to suppress glycolysis and proliferation in GBM cells by encoding HEATR5B-881aa [13]. Specifically, circRNAs or the selective enrichment of subtypes of circRNAs that are either lowly expressed or absent in exosomes from non-tumor cells may show a marked elevation in exosomes. Although circRNAs are prevalent in exosome secretion by various cell populations, tumor-derived exosome samples often have different circRNA profiles than those derived from normal cells [14–20]. Due to circRNAs’ extensive distribution and critical roles in tumor development, there is significant potential for exploring novel cancer immunotherapy approaches.

## Functional mechanism

The latest research indicates that circRNAs may have a multifaceted role in tumor development. Figure S1 delineates the functional mechanisms of circRNAs. The following section outlines the principal mechanisms.

### Acting as miRNA sponge

CircRNAs can sequester microRNAs (miRNAs), preventing them from binding to corresponding messenger RNAs (mRNAs). This results in the upregulation of downstream mRNA expression [21–23]. This is the most well-known mechanism. In every research project investigating circRNA mechanisms, this possibility is typically considered first. For example, Fei et al. demonstrated that circACVR2A significantly inhibits the proliferation and metastasis of hepatocellular carcinoma by directly binding to miR-511-5p. This reduces the inhibitory capacity of miR-511-5p on the PI3K signaling pathway [23]. However, it should be noted that not all circRNAs possess a miRNA-binding site; they may have other functions as well [6].

### Interacting with proteins

Some circRNAs with protein-binding sites can interact with specific proteins, which stabilizes them or prevents other molecules from binding to them [24–28]. For example, Du et al. found that circNFIB inhibited the proliferation and metastasis of intrahepatic cholangiocarcinoma (ICC) cells in vitro and in vivo. Further study revealed that circNFIB competitively interacts with MEK1, inducing the dissociation of MEK1 and ERK2 and resulting in the suppression of ERK signaling and tumor metastasis [25]. Additionally, numerous circRNAs have been shown to regulate protein degradation by modulating protein ubiquitination and deubiquitination. Yang et al. demonstrated that hypoxia-induced circWSB1 can physically bind to the deubiquitinase USP10, eliminating the interaction between USP10 and its target protein, p53. This results in the poly-ubiquitination and subsequent degradation of the p53 protein under hypoxic conditions, promoting breast cancer progression (BC) [28].

### Acting as a scaffold

Some circRNAs possess multiple protein-binding sites, enabling them to act as protein scaffolds and facilitate protein interactions [14, 29, 30]. For example, Xu et al. demonstrated that circPOLR2A can interact with UBE3C and PEBP1 proteins. UBE3C functions as a specific ubiquitin E3 ligase for the PEBP1 protein. The UBE3C/circPOLR2A/PEBP1 protein-RNA complex has been shown to increase the ubiquitination and degradation of PEBP1, a process mediated by UBE3C. Furthermore, this complex has been shown to activate the ERK pathway, which plays a role in the progression and metastasis of clear cell renal cell carcinoma (ccRCC) [29]. Ling et al. identified a circRNA, circCDYL2, that was highly expressed in patients who had developed trastuzumab resistance. This resulted in trastuzumab resistance in breast cancer cells. Further studies demonstrate that circCDYL2 stabilizes GRB7 by preventing its ubiquitination and degradation and enhancing its interaction with FAK. This maintains the activities of the downstream AKT and ERK1/2 pathways [30]. Additionally, circRNAs’ presence of both protein and mRNA-binding sites enables them to act as a scaffold between mRNA and protein [31].

### Templates for translation

Both circRNAs and their linear counterparts, mRNAs, originate from pre-mRNAs. Notably, the majority of circRNAs retain exons. While most circRNAs are noncoding, some have been shown to be capable of being translated into proteins [13, 32, 33]. IRES sequences have been identified as a means of cap-independent translation [1], and m6A modification has been demonstrated to play a pivotal role in promoting translation. CircRNA translation can be initiated as long as one of these two factors is present. Furthermore, the presence of both factors has been shown to enhance transcription efficiency [7, 34]. For example, circTRIM encodes the TRIM1-269aa protein, which has been associated with chemoresistance and TNBC metastasis. This process involves enhancing CaM-dependent MARCKS translocation and PI3K/AKT/mTOR activation [32]. Additionally, a novel protein encoded by circINSIG1 has been identified as a key player in reprogramming cholesterol metabolism by promoting the ubiquitin-dependent degradation of INSIG1 in colorectal cancer [33].

### Regulating translation

CircRNAs have been shown to regulate translation in several ways. For example, circ-ZNF609 interacts with several mRNAs, including CKAP5, UPF2, and SRRM1. It controls their stability and translation by recruiting the ELAVL1 RNA-binding protein. ELAVL1 is a highly sought-after protein, with numerous mRNAs competing for its binding. Therefore, CKAP5, which has a binding site only in the 3″ UTR, must interact with ELAVL1 with the help of circ-ZNF609 to facilitate translation [31]. Additionally, some circRNAs are capable of binding not only to mRNA but also to eukaryotic initiation factors (eIFs), thereby regulating translation through protein-binding sites [15, 35]. Wu et al. initially identified that circYap binds to Yap mRNA and translation initiation-associated proteins eIF4G and PABP. This complex results in the termination of the interaction between PABP on the poly(A) tail and eIF4G on the 5’-cap of Yap mRNA. This results in the suppression of Yap translation initiation [35]. Zheng et al. discovered that exosomal circLPAR1 directly binds to eIF3h, which specifically suppresses the METTL3-eIF3h interaction and decreases the translation of the oncogene BRD4 [15].

### Regulating transcription

CircRNAs can bind to target promoters and recruit the necessary proteins for transcription, thereby regulating it. Chen et al. demonstrated that circREEP3 initiates FKBP10 transcription by recruiting CHD7, a chromatin remodeler that regulates gene transcription. Furthermore, the loop structure of circREEP3 was shown to be essential for its interaction with CHD7, suggesting that the unique loop structure itself is also crucial in addition to the sequence [12]. Additionally, another study revealed that EIciRNAs can bind to U1 snRNP via RNA-RNA interaction. Subsequently, EIciRNA-U1 snRNP complexes may interact with the Pol II transcription complex at promoters of parental genes, enhancing gene expression [36].

Some circRNAs have been shown to perform additional functions, such as involvement in rRNA processing and pseudogene generation [2]. Furthermore, a single circRNA may have multiple functions. For example, circACTN4 recruits YBX1 to co-activate FZD7 transcription and upregulates YAP1 expression by sponging miR-424-5p [21].

## CircRNAs in Cancer progression

**Fig. 2:**
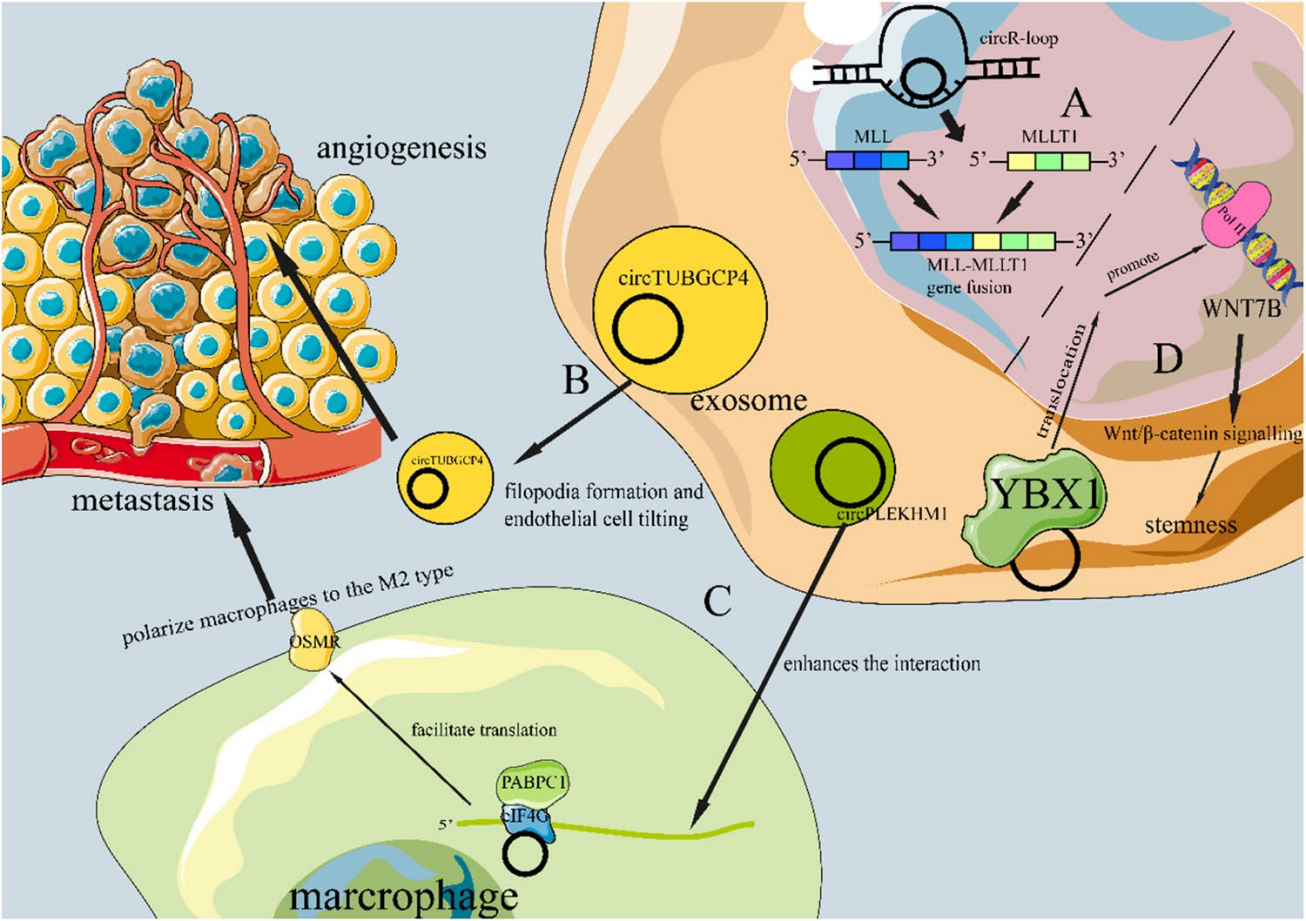
This picture delineates several circRNA function examples to show the relationship between circRNAs and tumor progression. **A.** CircR-loop induces oncogene formation via chromosomal translocation by inhibiting proteasomes and causing DNA double-strand breaks. **B.** CircTUBGCP4 in CRC cell exosome promotes vascular endothelial cell migration and tube formation by facilitating filopodia formation and endothelial cell tilting [17]. **C.** Exosomal circPLEKHM1 enhances the interaction between PABPC1 and eIF4G to facilitate oncostatin M receptor (OSMR) translation, thus promoting macrophage polarization inducing cancer metastasis [19]. **D.** CircRREB1 promotes WNT7B transcription by interacting with YBX1 and facilitating its nuclear translocation, thereby activating the Wnt/β-catenin pathway to maintain PDAC stemness [52]

Cancer development is a complex pathological process comprising numerous hallmarks, including genome instability and mutation, sustaining proliferative signaling, attaining replicative immortality, evading growth suppressors, withstanding cell death, activating invasion and metastasis, triggering angiogenesis, enhancing tumor inflammation and evading immune destruction [37]. Emerging evidences suggest that many circRNAs are closely associated with cancer development. Figure 2 delineates several circRNA function examples to show the relationship between circRNAs and tumor progression. Table 1 summarizes key circRNAs involved in various aspects of cancer progression.

**Table 1:** Overview of dysregulated circRNAs and their functions in cancer

Tumor type	CircRNA	Dysregulation	Function	Sample	Reference
Hepatocellular carcinoma (HCC)	circMDK	Up	The sponging of miR-346 and 874-3p upregulates the expression of autophagy-related protein 16-like 1 (ATG16L1), which activates the PI3K/AKT/mTOR signaling pathway and promotes cancer cell proliferation, migration, and invasion	HCC and matched adjacent noncancerous liver (ANL) tissues from HCC patients	[109]
	circACVR2A	Up	It interacts with miR511-5p and acts as a microRNA sponge, regulating the expression of related proteins in the PI3K-Akt signaling pathway	Huh-7, HepG2, Hep3B,HEK-293 cells and MIHA cells	[23]
	circPAK1	Up	It enhances HCC progression by competitively binding to 14–3-3 zeta (14–3-3ζ) with YAP, thereby promoting YAP nuclear localization and leading to the inactivation of the Hippo signaling pathway. CircPAK1 can be transported by exosomes from lenvatinib-resistant cells to sensitive cells, inducing lenvatinib resistance	HCC tissue samples from HCC patients. Human HCC cell lines, including HepG2, Focus, Hep3B, MHCC-97 L, HCC-LM3 (LM3), YY8103, Huh-7, and L02 cell lines	[42]
	circPSD3	Down	Inhibit the intrahepatic vascular invasion and metastasis of HCC cells by interacting with histone deacetylase 1 (HDAC1) and sequestering it in the cytoplasm. This attenuates the inhibitory effect of HDAC1 on the transcription of serpin family B member 2 (SERPINB2)	HCC samples from HCC patients. Human HCC cell lines (HCC-LM9 and SK-Hep-1) cells	[110]
Bladder cancer	circSTX6	Up	It promotes cell migration and invasion by acting as a sponge for miR-515-3p and abolishing its effect on SUZ12. Furthermore, circSTX6 stabilizes SUZ12 mRNA by interacting with the mRNA stabilizer PABPC1, thereby promoting SUZ12 expression	bladder cancer tissues and NATs from patients. Human normal bladder epithelial cell line SV-HUC-1 was purchased from ATCC. The human urinary bladder transitional cell carcinoma cell lines T24, RT4, UMUC3 and 5637.EJ cells	[111]
Gastric cancer (GC)	circ0001947	Up	Sponge miR-661 and miR-671-5p to increase CD39 expression, which facilitates CD8 + T cell exhaustion and immune resistance	The human gastric epithelial cell line GES − 1, human GC cell line AGS, and murine GC cell line MFC	[63]
	circARID1A	Up	It serves as a scaffold that facilitates interaction between IGF2BP3 and SLC7A5 mRNA, thereby increasing SLC7A5 mRNA stability. Additionally, circARID1A can directly bind to SLC7A5 mRNA through complementary base pairing. Then, it forms a circARID1A-IGF2BP3-SLC7A5 RNA–protein complex, which promotes the proliferation of GC cells by regulating the AKT/mTOR pathway	GC and the corresponding noncancerous tissue samples from patients	[112]
Lung cancer	circPLEKHM1	Up	Promote the interaction between PABPC1 and eIF4G to facilitate the translation of the oncostatin M receptor (OSMR). This will promote macrophage polarization, which is necessary for cancer metastasis	Human monocyte/macrophage cell line THP‐1, human non‐small cell lung cancer cell lines A549 and H1299. Human lung cancer cell line PC9 and mouse lung cancer cell line LA795. Human bone mesenchymal stem cells (MSCs)	[19]
	circNOX4	Up	Upregulating FAP by sponging miR-329-5p leads to fibroblast activation. This activates an inflammatory fibroblast niche by preferentially inducing interleukin-6 (IL-6), which eventually promotes NSCLC progression	Tumor samples from NSCLC patients	[71]
	circZNF451	Up	Reshape the tumor immune microenvironment by polarizing macrophages via the FXR1-ELF4-IRF4 axis	LUAD cell lines A549, H1299, Calu-3, H1975, H1395, and LLC, HEK-293 T cells, and THP-1 cells	[14]
	circFIRRE	Up	It drives primary osteosarcoma progression and lung metastasis by increasing the mRNA and protein levels of LUZP1 through the sponging of miR-486-3p and miR-1225-5p	OS samples and their corresponding adjacent samples from patients	[43]
	circHMGB2	Up	Relieve the inhibition of CARM1 downstream by sponging miR-181a-5p to inactivate the type 1 interferon response in lung adenocarcinomas (LUAD) and squamous cell carcinomas (LUSC)	human NSCLC cell lines A549, PC9, NCI-H460, NCI-H1299, NCI-H1703 and human bronchial epithelial (HBE) cells. The mouse lung cancer cell lines LLC and HEK-293 T cells	[64]
Glioblastoma (GBM)	circHEATR5B	Down	Encode the novel protein HEATR5B-881aa, which interacts directly with Jumonji C-domain-containing 5 (JMJD5) and reduces its stability through the phosphorylation of S361	human glioma tissues, human GBM cell lines (U87, U251, U373, and A172) and 293 T cells, Normal human astrocytes (NHAs)	[13]
	circCMTM3	Up	Activate the JAK2/STAT5A pathway in a non-canonical manner to promote the formation of vasculogenic mimicry (VM) in GBM	glioma tissue specimen	[18]
Prostate cancer (PCa)	circPDE5A	Down	Form the circPDE5A-WTAP complex to block the WTAP-dependent N6-methyladenosine (m6A) methylation of eukaryotic translation initiation factor 3C (EIF3C) mRNA and finally disrupt the translation of EIF3C	prostate cancer samples, Human prostate cancer cells (LNCaP, C4-2B, 22Rv-1, DU145, and PC-3), and human normal prostate epithelial RWPE-1 cells	[24]
Colorectal cancer (CRC)	circREEP3	Up	Stimulate PGK1 to induce glycolysis and activate the Wnt/β-catenin signaling pathway in order to maintain stemness in pancreatic cancer	CRC samples and Colorectal cancer cell lines	[12]
	circEXOC6B	Down	Inhibit CRC progression and enhance CRC cell chemosensitivity to 5-fluorouracil by antagonizing the HIF1A-RRAGB-mTORC1 positive feedback loop	Human CRC cell lines SW480, SW620, RKO, Caco-2, HCT116, LoVo and HT29. CRC tissues and adjacent noncancerous tissues	[26]
	circLPAR1	Down	Binding directly to eIF3h suppresses the METTL3-eIF3h interaction, thereby decreasing the translation of the oncogene BRD4	patients with gastric carcinoma, breast invasive carcinoma, bladder urothelial carcinoma, cervical squamous cell carcinoma and endocervical adenocarcinoma, kidney renal clear cell carcinoma, and lung adenocarcinoma, their corresponding tumor/normal tissues and peripheral blood samples and plasma samples from colorectal cancer patients	[15]
	circINSIG1	Up	The circINSIG1-121 protein is encoded to promote K48-linked ubiquitination of the critical cholesterol metabolism regulator INSIG1 at lysines 156 and 158. This is done by recruiting the CUL5-ASB6 complex, which is a ubiquitin E3 ligase complex. This process induces cholesterol biosynthesis, thereby promoting CRC proliferation and metastasis	human CRC cell lines (HCT8, DLD1, HCT116, SW620, CaCO2 and HT29), normal human intestinal epithelial cell lines (HIEC-6) and human embryonic kidney 293T cells. Intestines and livers from mouse	[33]
	circPOLQ	Up	Promote M2 macrophage polarization by activating the interleukin-10/signal transducer and activator of transcription 3 axis through targeting miR-379-3p	CRC tissues and matched adjacent normal tissues. HCT116, LoVo, HT29, SW620, FHC, THP-1, and 293T cell lines	[20]
Pancreatic cancer	circANAPC7	Down	The CREB-miR-373-PHLPP2 axis functions to suppress tumor growth and muscle wasting in pancreatic cancer by leading to AKT dephosphorylation and the downregulation of cyclin D1 and transforming growth factor-β	Human pancreatic cancer cell lines AsPC-1, MIA PaCa-2, BxPC-3, Panc-1, and CFPAC-1. Murine C2C12 myoblasts. human pancreatic cancer tissue	[72]
	hsa_circ_0007919	Up	Maintain cell survival by enhancing DNA damage repair in a LIG1-dependent manner	PDAC tissues and adjacent tumor tissues	[113]
	circPDK1	Up	It is activated by HIF1A at the transcriptional level, which then modulates the miR-628-3p/BPTF axis and degrades BIN1	Inhibit proliferation, migration, and glycolysis. Normal human pancreatic duct cell line hTERT-HPNE and PDAC cell lines PANC-1, CFPAC-1, BxPC-3 and MIA-PaCa2	[94]
Breast cancer	circRNA-CREIT	Down	It acts as a scaffold that facilitates the interaction between PKR and the E3 ligase HACE1, thereby promoting the proteasomal degradation of PKR protein. Reduced PKR/eIF2α signaling attenuates stress granule (SG) assembly, activating the RACK1/MTK1 apoptosis signaling pathway	tissue samples from patients. HEK-293T cells, the human breast cancer cell lines MDA-MB-231, MDA-MB-468, MDA-MB-436, MCF-7, ZR-75–1, SKBR3 and HS578T, and the human normal mammary epithelial cell line MCF10A	[57]
	circCDYL2	Up	Stabilize GRB7 by preventing its ubiquitination and degradation, and enhancing its interaction with FAK. This will sustain the activities of the downstream proteins AKT and ERK1/2	BC patient tissues	[30]
Cervical cancer	circ_0087429	Down	Upregulate the expression of OGN by competitively binding to miR-5003-3p. This will reverse epithelial-to-mesenchymal transition (EMT) and inhibit the progression of cervical cancer	cervical cancer tissues and matched adjacent tissues. human cervical immortalized squamous cell line (Ect1/E6E7) and human cervical cancer cell lines (HeLa, SiHa and CaSki)	[114]
Osteosarcoma	circFIRRE	Up	Increase the mRNA and protein levels of LUZP1 by sponging miR-486-3p and miR-1225-5p	OS samples and their corresponding adjacent samples	[43]
	circKEAP1	Down	Encode the truncated KEAP1-259aa protein, which binds to vimentin in the cytoplasm and promotes its proteasomal degradation by interacting with the E3 ubiquitin-protein ligase ARIH1	OS tissues and normal adjacent tissues. HEK-293T human embryonic kidney cells, the hFOB1.19 osteoblast cell line, and OS cell lines U2OS, Saos-2, HOS and MG63	[51]

### CircRNAs regulating genome instability and mutation

The initial acquisition of a repertoire of genetic mutations is the first step in oncogenesis, the process by which these mutations initiate and sustain malignant cell growth. Specific mutant genotypes confer a selective advantage to subclones of cells, facilitating their growth and eventual predominance in a local tissue [37]. A recent study by Conn et al. found evidence that some circRNAs influence this process. Chromosomal translocations between the mixed lineage leukemia (MLL) gene and its translocation partners—collectively known as the MLL recombinome—are a primary initiating event in acute leukemias. The study showed that circMLL(9,10) is common in the MLL recombinome and can bind to DNA to form circRNA:DNA hybrids (circR loops) at their respective locations. Formation of circR loops results in transcriptional pausing and proteasome inhibition. Prolonged formation of DNA:RNA:DNA, resulting from transcriptional pausing, leaves single-stranded DNA (ssDNA) susceptible to gene mutation. Meanwhile, the inhibited proteasome cannot repair the double-strand DNA breaks caused by base excision repair to the ssDNA [29, 30, 38]. Furthermore, circMLL(9,10) has been shown to drive oncogenic translocations in vitro and in vivo and accelerate the onset of leukemia in mice [38].

P53, also known as TP53, plays an oncogenic role when mutated and has a high mutation rate in tumor patients [2, 39]. Liu et al. conducted a study investigating the effects of continuous exposure to the tobacco-specific carcinogen 4-(methylnitrosamino)-1-(3-pyridyl)-1-butanone (NNK) on bronchial epithelial cells, which led to malignant transformation. They focused on CircNIPBL, which was found to promote the accumulation of TP53-H179R during NNK-induced carcinogenesis by interacting with HSP90α to control AHR translocation into the nucleus [40].

Some circRNAs do not directly cause genomic mutations, but they can interact with the expression products of mutated genes [2]. Wang et al. found that circCFL1 acts as a scaffold that enhances the interaction between HDAC1 and c-Myc. This further promotes c-Myc stability through deacetylation-mediated inhibition of K48-linked ubiquitylation. Stably expressed c-Myc then increased the expression of mutp53 in triple-negative breast cancer (TNBC) cells with TP53 mutations by binding directly to the TP53 promoter. This increases the stemness of TNBC cells by activating the p-AKT/WIP/YAP/TAZ signaling pathway [39].

In general, circRNAs play an important role in chromosome translocation, gene mutation, and interaction with mutated genes in tumors.

### CircRNAs and angiogenesis

Tumors require nutrients and oxygen. Angiogenesis, or the formation of new blood vessels, is particularly relevant in tumor conditions [37, 41]. CircRNAs have been demonstrated to play significant roles in angiogenesis [42–45]. Chen et al. reported that the upregulation of circTUBGCP4 in exosomes from CRC cells enhances vascular endothelial cell migration and tube formation by inducing filopodia formation and endothelial cell tilting. They also found that circTUBGCP4 increased PDK2 expression, thereby activating the Akt signaling pathway by sequestering miR-146b-3p [17]. In addition, vascular endothelial growth factor A (VEGF-A), a well-known inducer of angiogenesis, is closely related to some circRNAs [37]. Li et al. reported that the upregulation of circFNDC3B in oral squamous cell carcinoma (OSCC) controls the ubiquitylation of the RNA-binding protein FUS and the deubiquitylation of HIF1A via the E3 ligase MDM2, thereby enhancing VEGF-A transcription and promoting angiogenesis [46]. Hypoxia is also a notable stimulus for angiogenesis [47]. Mao et al. found that hypoxic exosomes from esophageal squamous cell carcinoma (ESCC) disrupted the tight junctions of human umbilical vein endothelial cells (HUVECs) and enhanced the expression of angiogenesis-related proteins. Meanwhile, hypoxia remarkably induced circ-ZNF609 expression in ESCC exosomes, which were internalized by HUVECs, as determined by circular RNA screening. High levels of circ-ZNF609 in HUVECs facilitate angiogenesis and vascular permeability. This process promotes pre-metastatic niche formation and enhances distant metastasis in both in vitro and in vivo models. Circ-ZNF609 activates VEGF-A by sequestering miR-150-5p [44]. In glioma, there is an increase in the expression of circ_0059914. The study by Hedayati et al. demonstrates that circ_0059914 promotes angiogenesis. Glioma cell tube formation is impaired upon knockdown and enhanced upon overexpression. Circ_0059914 functions as a sponge for miR-1249, alleviating the repression of VEGFA mediated by miR-1249. Furthermore, EIF4A3 can induce circ_0059914 biogenesis. In an in vivo setting, circ_0059914 expression has been observed to promote xenograft growth by enhancing angiogenesis, while knockdown suppresses this process [41]. In non-small cell lung cancer (NSCLC), circ_0101675 (derived from NPAS3) expression is notably elevated. Studies have demonstrated that circ_0101675 stimulates angiogenesis. Silencing its gene expression reduces the formation of blood vessels by NSCLC-induced HUVECs, while expressing circ_0101675 promotes this process. Circ_0101675 functions as a sponge for miR-607, thereby upregulating PD-L1. This process enhances angiogenesis [48].

### CircRNAs and metastasis

Carcinomas that originate in epithelial tissues tend to progress to more severe pathological grades of malignancy, as indicated by local invasion and distant metastasis [37]. Indeed, many circRNAs have been found to be associated with metastasis. For instance, Xi et al. found that circBCAR3 is highly expressed in esophageal cancer tissue and binds to miR-27a-3p, thereby increasing TNPO1 expression. Overexpression of TNPO1 reversed circBCAR3-mediated suppression of esophageal cancer cell proliferation, migration, invasion, and ferroptosis [49]. Through systematic analysis of exosomes secreted by NSCLC cells, Wang et al. discovered a hypoxia-induced exosomal circPLEKHM1. This circPLEKHM1 drives NSCLC metastasis by polarizing macrophages to the M2 type. Mechanistically, exosomal circPLEKHM1 enhances the interaction between PABPC1 and 4G, facilitating the translation of OSMR and promoting macrophage polarization for cancer metastasis. Furthermore, circPLEKHM1-targeted therapy significantly reduces NSCLC metastasis in vivo [19].

### CircRNAs and stemness

Stemness plays a critical role in tumorigenesis, development, drug resistance, metastasis, and immune escape. Zhang et al. demonstrate that circKEAP1 acts as a tumor suppressor in osteosarcoma (OS) and exhibits low expression levels in OS. CircKEAP1 encodes the KEAP1-259aa protein, which binds to vimentin and contributes to stem-like properties in MDA-MB-231 breast cancer cells [50]. CircKEAP1 also promotes vimentin degradation by interacting with the E3 ligase ARIH1. Consequently, circKEAP1 suppresses cancer stemness, invasion, and proliferation [51]. Another study by Rong et al. revealed that circRREB1 promotes WNT7B transcription by directly interacting with YBX1 and facilitating its nuclear translocation. This activates the Wnt/β-catenin signaling pathway, thereby maintaining PDAC stemness [52]. As previously mentioned, circular RNA CFL1 has been identified as being upregulated in TNBC and acting as a tumor stemness maintainer [39]. These findings suggest that circRNAs exhibit a high degree of correlation with stemness formation.

### CircRNAs and chemical drug resistance

Chemoresistance remains a major challenge in clinical practice. Recent studies have shown that circular RNAs (circRNAs) play a critical role in chemoresistance [39, 53–57]. Pan et al. found that circATG4B was upregulated in exosomes secreted by oxaliplatin-resistant colorectal cancer (CRC) cells. CircATG4B encodes the protein circATG4B-222aa, which acts as a decoy by competitively interacting with TMED10. This prevents TMED10 from binding to the autophagy-related protein ATG4B. Consequently, autophagy increases, leading to the induction of chemoresistance [56]. Wang et al. demonstrated that overexpressing circRNA-CREIT significantly increased the sensitivity of TNBC cells to doxorubicin. Mechanistically, circRNA-CREIT acts as a scaffold that facilitates interaction between PKR and the E3 ligase HACE1 (HECT domain- and ankyrin-repeat-containing E3 ubiquitin-protein ligase 1), promoting the proteasomal degradation of PKR protein via K48-linked polyubiquitylation. A reduced PKR/eIF2α signaling axis was identified as a critical downstream effector of circRNA-CREIT. This reduced signaling axis attenuates the assembly of stress granules (SGs) and activates the RACK1/MTK1 apoptosis signaling pathway. Additionally, circRNA-CREIT can be packaged into exosomes and spread doxorubicin sensitivity among TNBC cells [57].

## CircRNAs and Immunotherapy

Although tumor immunotherapy has made much progress [58], there are still many limitations, such as drug resistance and low response rates [59, 60]. Cancer is now seen as an ongoing process involving constant interactions between cancer cells and the tumor microenvironment (TME). The TME is made up of all the noncancerous host cells in the tumor and non-cellular components. Some of these components are the extracellular matrix (ECM) and soluble products such as chemokines, cytokines. Many immunotherapies yield limited results under the influence of the tumor microenvironment [61]. Recent studies show that circRNAs may play a key role in immunotherapy and TME reshaping. The unique property may help us overcome the limitations. Figure S2 explains several therapeutic strategies utilizing circRNAs in cancer, covering circRNA-based vaccines, CAR-T, and targeting approaches.

### CircRNAs in immune checkpoint blocking (ICB)

In recent years, ICB therapy, specifically antibodies against PD-1/PD-L1 or CTLA-4 signaling, has been widely used in cancer treatment [59], which has significantly prolonged the survival of cancer patients. However, the partial or complete response rate is low due to T cell exhaustion and TME [62]. Recently, some studies have reported circRNAs that regulate anti-ICB resistance [62–66]. Hu et al. reported that CD8 + T cells exhibited greater cytotoxicity to hepatocellular carcinoma (HCC) cells and generated more cytokines when incubated with exosomes from HCC cells in which circCCAR1 was knocked down. Functionally, circCCAR1 prevented PD-1 from ubiquitination-mediated degradation by directly binding to PD-1. They also observed that the mRNA and protein levels of CCAR1, the host gene of circCCAR1, were upregulated in HCC [62]. The increased CCAR1 expression enhanced PD-L1 expression levels by interacting with β-catenin, further weakening the immune response of HCC [62]. Similarly, Zang et al. discovered that circPIAS1 is capable of encoding a unique polypeptide consisting of 108 amino acids. Through this polypeptide, circPIAS1 exerts its cancer-promoting role and inhibits the efficacy of ICB therapy. In a further study, they investigated the impact of circPIAS1 on immunotherapy. Specific antisense oligonucleotides (ASOs) were designed to knock down circPIAS1, and the result shows that blocking circPIAS1 significantly increases the efficacy of PD-1 inhibitors [66]. Apart from PD-1/PD-L1 inhibitors, CTLA-4 inhibitors are also related. Liu et al. reported that m6A-modified circQSOX1 promoted CRC tumor development by binding to miR-326 and miR-330-5p, thereby enhancing the expression of PGAM1. This subsequently promoted CRC immune escape by activating glycolysis and disabling the anti-CTLA-4 therapy response to CRC [65]. CircRNAs in TME regulation are also very important. Zhang et al. found that circHMGB2 exerts a modest influence on the proliferation of the lung adenocarcinomas (LUAD) and squamous cell carcinomas (LUSC). However, circHMGB2 significantly impacts the tumor microenvironment by contributing to the exhaustion of antitumor immunity in both an immunocompetent mouse model and a humanized mouse model. The study demonstrated that the intervention led to the inhibition of IFN response-related gene expression and the enhancement of tumor resistance to cytotoxic T cells. This, in turn, resulted in the induction of an immunosuppressive microenvironment in NSCLC. It inhibited IFN response-related gene expression and enhanced tumor resistance to cytotoxic T cells. This further induced an immunosuppressive microenvironment in NSCLC [64]. It is posited that if we manage to downregulate the associated circRNAs or block their function, ICB therapy would have a better effect toward solid tumor.

### CircRNAs and cytokine

Cytokines are critical in regulating immune responses and cellular behavior [67]. Several cytokines, including IL-2, IL-12 and IFN-α, have antitumor activity [68]. In contrast, certain cytokines can be co-opted to promote cancer progression [69] such as TGF-β [69] and IL-6 [70]. CircRNAs have been found to play a pivotal role in cytokine secretion [20, 71–74].

TGF-β plays two distinct roles. Initially, it inhibits tumor formation by arresting the cell cycle. But in later stages, it helps tumor growth by facilitating EMT, promoting metastasis, chemoresistance, angiogenesis and immune evasion [75]. Recent studies show that circRNAs interact with TGF-β. Shi et al. have identified circANAPC7 as a novel tumor suppressor. It acts through the CREB-miR-373-PHLPP2 axis, resulting in AKT dephosphorylation and reduction of cyclin D1 and TGF-β to inhibit tumor progression in pancreatic cancer [72]. In addition, Li et al. discovered a TGF-β-induced circRNA called circITGB6 as an essential factor in the TGF-β-mediated EMT process. In terms of mechanism, circITGB6 increases the mRNA stability of PDPN, a gene that promotes EMT, by directly interacting with IGF2BP3 and interfering with circITGB6 with PEI-coated specific siRNA effectively suppresses liver metastasis [74].

Dysregulation of IL-6 signaling not only directly promotes tumor growth, but also contributes significantly to immune evasion by modifying TME [69, 70]. Li et al. detected the differentially expressed circRNAs when IL-6 stimulated ICC cells and found that circGGNBP2 was upregulated by IL-6 treatment. The formation of circGGNBP2 was regulated by the RNA-binding protein DEx-H box helicase 9, which was also mediated by IL-6 exposure. CircGGNBP2 encoded a protein named cGGNBP2-184aa and cGGNBP2-184aa directly interacted with signal transducers and activators of transduction-3 (STAT3) and phosphorylated STAT3Tyr705, playing a positive regulatory role in modulating IL-6/STAT3 signaling. IL-6/cGGNBP2-184aa/STAT3 formed a positive feedback loop to maintain the continuous activation of IL-6/STAT3 signaling. Elevated cGGNBP2 expression was correlated with TME maintaining and poor prognosis in patients with ICC [76]. Another research conducted by Zhao et al. shows that circNOX4 upregulates fibroblast activation protein (FAP) by sponging miR-329-5p in NSCLC. The circNOX4/miR-329-5p/FAP axis activated an inflammatory fibroblast niche by preferentially inducing IL-6 and ultimately promoting NSCLC progression. Disruption of the intercellular circNOX4/IL-6 axis significantly suppressed tumor growth and metastatic colonization in vivo [71].

### CircRNAs as cancer vaccine

A tumor vaccine induces a new or enhanced immune response of CD8 + cytotoxic T cells (CTL) by introducing tumor-specific antigens into the human body [7]. In the course of cancer development, genomic instability, such as gene mutations in the coding region, causes a change in the protein sequence. The newly produced protein in cancer cells while common cells don’t have is called a neoantigen. Neoantigens can be displayed on the cell surface by major histocompatibility complexes (MHC) and subsequently recognized by T cells to trigger the immune response [7]. Tumor vaccines targeting neoantigens mainly include nucleic acid, dendritic cell (DC)-based, tumor cell and synthetic long peptide (SLP) vaccines [77]. Among these types of cancer vaccines, RNA-based vaccines, especially mRNA vaccines, are particularly attractive. They can rapidly express antigens in the cytoplasm and induce strong immune activation. In addition, they have the advantage of avoiding the risks associated with genome integration and T cell tolerance [78]. However, mRNA vaccines have poor thermal stability and are easily degraded when administered in vivo [7]. In this context, the characteristics of circRNAs can be readily considered. By integrating the protein-coding ability and extremely high stability of circRNAs, it is possible to use circRNAs encoding neoantigens as a new type of tumor vaccine. Wang et al. performed a comparative experiment between linear mRNA vaccines and circRNA vaccines. Compared with the PBS and linear RNA (linRNA) treatment groups, the circRNA treatment group significantly reduced the luciferase activity at the tumor site with prolonged treatment time due to the excellent therapeutic effects. They also found that mice receiving the circRNA vaccine had a prolonged overall survival time compared to the PBS or linRNA groups. In addition, no apparent toxic side effects were observed. [78] Ren et al. reported the potential of circular RNA as a source of neoantigens for cancer vaccines. Their study shows that circRNAs are a source of neoantigens and that neoantigens derived from circRNAs are immunogenic. In addition, neoantigens from differentially expressed circRNAs in patients with CRC drive tumor-specific killing in patient-derived organoids, proving that circRNAs can act as a cancer vaccine [79]. However, researches in this area are just beginning and further investigation is needed.

### CircRNAs and in vivo CAR-T therapy

CAR-T represents a significant advancement in the field of tumor immunotherapy. However, this treatment remains a number of serious side effects, including cytokine-release syndrome and on-target/off-tumor effects [60, 80]. And most CAR-T cells are produced by viral vector-based CAR engineering technologies. It is inevitable that these technologies will result in side effects associated with the vector and risks from genome integration and the permanent CAR expression on T cells [81]. Significant efforts have been made to develop alternative non-viral platforms. Among them mRNA-based CAR expression has distinctive advantages, including transient protein expression and non-viral delivery platforms [82, 83]. However, linear mRNAs are inherently unstable within the body, which inevitably results in a short duration of efficacy [84]. In contrast, circular mRNAs (cmRNAs), which are covalently closed circular RNA molecules, possess greater stability compared to linear mRNAs [85].

Orna Therapeutics has developed a novel circular coding RNA platform, designated orna RNA (oRNA) technology. They combine oRNA technology with immunotropic LNPs and create “autologous” in situ CAR (isCAR™) therapies. The study indicated that the immunotropic LNPs have a preferential biodistribution to the spleen and are capable of delivering oRNA to multiple immune cell subsets in different animals. In vitro, human T cells expressing anti-human CD19 CAR oRNA have been observed to demonstrate potent and sustained cytotoxicity and cytokine production. Additionally, the team developed the Formulated oRNA Cell-based Evaluation (FoRCE) platform, which facilitates high-throughput synthesis, purification, LNP formulation, and cell-based screening of oRNAs. Through screening, hundreds of IRESs were identified, with selected ones demonstrating high CAR expression and cytotoxicity in primary human T cells. The process of optimisation resulted in a 20-fold increase of efficacy in a mouse model. The optimized LNP-oCAR enables weekly dosage at clinically relevant levels with good tolerability and reproducible efficacy. It was hypothesized that oRNA-enabled isCAR therapies offer a promising transient, re-dosable and scalable immune cell therapy for cancer treatment.93.

Hu et al. devised a novel permuted intron–exon (PIE) strategy for the synthesis of cmRNA, which possesses both scarless and high-yield capabilities. These characteristics are of paramount importance for the development of drugs, as they facilitate the druggability of the target. The authors demonstrated that cmRNA augmented the amount and lengthened the duration of CAR expression on human T cells and illustrated the in vitro and in vivo merits of cmRNA in CAR expression and CAR-T therapy with an approved anti-CD19-CAR construct. The cmRNA-based anti-CD19 CAR-T cells exhibited augmented intensity and a prolonged duration of CAR expression on primary human T cells. Furthermore, the results demonstrated that primary human T cells transfected with CAR-encoding cmRNA exhibited enhanced potency in priming cytotoxic T cells in vitro compared to those transfected with linear mRNA. 94 Their work has demonstrated that the use of circRNAs in place of mRNA in CAR-T technology is both a practical and highly promising approach.

## CircRNAs as biomarker

Although certain biomarkers, such as PD-L1 expression and tumor mutational burden (TMB), have been associated with the response to tumor immunotherapy, these indicators have been found to lack complete accuracy. Notably, a significant proportion of patients with high PD-L1 expression or high TMB may not respond to immunotherapy. Conversely, patients with low expression or TMB may have unanticipated responses of notable efficacy. The absence of reliable predictive indicators complicates the selection of suitable patients for immunotherapy in clinical practice. This can result in the inefficient allocation of medical resources and the occurrence of adverse side effects [86, 87]. The close association of circular RNAs (circRNAs) with cancer development and progression, in conjunction with their differential expression between tumor and normal tissue, suggests their potential utilization. The abundance and stability of circRNAs contribute to their appeal. Their presence in various body fluids and enrichment in exosome content renders them suitable for liquid biopsies, a cutting-edge technology with considerable promise that has the potential to transform cancer screening and monitoring practices by providing a more precise and accessible alternative to conventional methods [3, 16, 88, 89].

### CircRNAs in diagnostics

CircRNAs have received significant attention as potential diagnostic biomarkers in oncology. Accumulating research has highlighted their usefulness in the early detection and classification of various cancers [15, 90–92].

Notably, Xu et al. investigated the diagnostic potential of a circRNA panel in pancreatic ductal adenocarcinoma (PDAC), a disease with a delayed clinical presentation and limited early detection tools. They found that a panel of five candidate circRNAs had high diagnostic accuracy, distinguishing PDAC patients from noncancerous controls and differentiating between early- and late-stage disease. The panel consistently produced high area under the curve (AUC) values in both the training and validation cohorts, especially for the detection of early-stage PDAC. This is a critical advantage given the prognostic significance of early diagnosis in this aggressive malignancy. A notable finding was that combining the circRNA panel with cancer antigen 19–9 (CA19-9) levels substantially enhanced overall diagnostic efficacy. This addresses a salient limitation of CA19-9 alone: its diminished sensitivity among specific patient subpopulations. The circRNA panel was highly effective in identifying PDAC cases that were clinically CA19-9 negative, a subgroup that often evades timely detection. These findings highlight the potential of circRNA-based panels as complementary or standalone tools for early and accurate PDAC diagnosis. These findings have implications for improving patient management and outcomes in this challenging disease [93].

### CircRNAs in prognosis

CircRNAs have emerged as pivotal regulators in cancer progression and are increasingly recognized as promising prognostic indicators. Accumulating evidence indicates that the dysregulated expression of specific circRNAs—whether due to upregulation or downregulation—is closely associated with poor clinical outcomes in various malignancies, highlighting their potential as robust prognostic biomarkers [12, 21, 22, 25, 55, 76, 94].

Extensive investigations by multiple research groups have illuminated the clinical significance of circRNAs in cancer prognosis. These studies have revealed that circRNAs can stratify patients into distinct risk groups and predict disease progression trajectories. For example, Zheng et al. demonstrated that circFOXK2 is significantly upregulated in HCC tissues compared to adjacent non-tumor tissues. From a clinical perspective, elevated circFOXK2 expression levels have been shown to correlate significantly with poor outcomes in HCC patients who have undergone radical hepatectomy, as evidenced by lower overall and disease-free survival rates. Their study revealed that circFOXK2 exerts its oncogenic effects through dual regulation: it promotes the expression of metabolic enzymes involved in the Warburg effect (a hallmark of cancer metabolism) and acts as a microRNA (miRNA) sponge to sequester tumor-suppressive miRNAs, thereby facilitating HCC progression. Taken together, these findings suggest that circFOXK2 is a valuable prognostic biomarker and potential therapeutic target in HCC [95]. In another notable study, Lin et al. found that circPDK1 is transcriptionally activated by hypoxia-inducible factor 1 alpha (HIF1A) under hypoxic conditions, a common feature of the tumor microenvironment. This study shows that circPDK1 modulates the miR-628-3p/BPTF axis, promoting the degradation of the tumor suppressor BIN1 and driving PC progression. Interestingly, the study revealed a correlation between serum levels of exosomal circPDK1 and aggressive clinicopathological features of PC. This correlation serves as an independent prognostic factor for poor survival. This work underscores the potential of exosomal circPDK1 as a noninvasive, clinically viable prognostic biomarker for PC, providing novel insights into liquid biopsy–based risk assessment in this refractory malignancy [94]. Taken together, these studies, in conjunction with a growing body of related literature, highlight the multifaceted roles of circRNAs in cancer biology and their potential as prognostic markers. These findings provide a rationale for further exploring their clinical utility in risk stratification and treatment decision-making.

## Limitations and future prospects

Despite their numerous advantages, circRNAs face many challenges in cancer therapy. In the context of cancer, many unknown elements remain within the intricate and specific regulatory networks in which circRNAs participate. The precise manner in which circRNAs influence diverse signal pathways in cancer cells and their relationship with other noncoding and coding RNAs remains unclear. Furthermore, circRNAs’ unique circular structure confers a remarkable degree of stability upon them. However, in the context of cancer treatment, this stability presents a duality. On the one hand, this phenomenon has the potential to accelerate the development of long-acting treatment agents. However, when the expression of circRNAs must be reduced or eliminated, their stability poses a significant challenge. Conventional methods for regulating their expression level and activity state in vivo are often imprecise. Additionally, enhancing the efficiency of circRNA delivery into cancer cells is necessary. Guaranteeing the delivery of sufficient quantities of circRNAs into cancer cells to elicit the desired outcomes is challenging.

Although many circRNAs have been shown to play a role in tumor formation, there is a lack of evidence supporting the specificity of individual circRNAs to a particular tumor type. Recent studies have revealed the multifaceted effects of circRNAs on the mechanisms involved in atherosclerosis development. These studies reveal that certain circRNAs can have opposing or contradictory effects on atherosclerosis, highlighting the complexity of their regulatory role in biological processes. Notably, circRNAs often contain multiple microRNA-binding sites on a single molecule, enabling interaction with various microRNA types. For a subset of circRNAs, the majority of their binding miRNAs exhibit synergistic effects, while a small fraction of miRNAs may exhibit conflicting or even diametrically opposed functions [96–99]. Conversely, a single miRNA may bind to multiple distinct circRNAs, resulting in outcomes that are diametrically opposed. Conversely, a single miRNA may bind to multiple distinct circRNAs, resulting in diametrically opposed outcomes [100–103]. For instance, circCRIM1 has been shown to impede osteosarcoma progression by sponging miR146a-5p. However, concurrent studies have indicated that circCRIM1 may promote nasopharyngeal carcinoma, osteosarcoma, and triple-negative breast cancer development [104–107]. This ambiguity makes certain circRNAs unsuitable for use as biomarkers due to their inability to exhibit specificity for cancer. As research progresses, this off-target phenomenon will likely become more prevalent. This phenomenon indicates the complex regulatory processes inherent in biological systems and underscores the necessity of meticulous analysis before initiating clinical trials. A comprehensive identification and mitigation strategy is imperative prior to the initiation of subsequent trials to address potential adverse effects [108].

According to clinicaltrials.gov, there are currently approximately a dozen clinical trials involving circRNAs. The majority of these trials are in a “not recruiting” status, and only three are actively recruiting participants. Table 2 provides an overview of ongoing circRNA-focused clinical trials. To date, no clinical data has been released. The potential for unintended adverse effects resulting from the long-term application of circRNAs in patients has yet to be elucidated. However, there are numerous avenues of investigation that remain to be explored in the field of circRNAs. Significant advancements are anticipated in the near future.

**Table 2:** Clinical trials involved in circRNAs

NCT Number	Study Title	Study Status	Conditions	References
NCT04584996	Circular and Noncoding RNAs as Clinically Useful Biomarkers in Pancreaticobiliary Cancers	UNKNOWN	Pancreatic Cancer|Biliary Tract Cancer	[115]
NCT05934045	Deciphering the Role of Circular RNAs in ALK positive Anaplastic Large-cell Lymphoma	ACTIVE_NOT_RECRUITING	ALK-Positive Anaplastic Large Cell Lymphoma	[116]
NCT06042842	The Value of circRNAs (hsa_circ_0004001) in Early Diagnosis of HCC	NOT_YET_RECRUITING	Hepatic Cell Carcinoma	[117]
NCT05771337	Circular RNA and Chemerin in Breast Cancer Patient	NOT_YET_RECRUITING	Breast Cancer	[118]
NCT06530082	A Single Arm Clinical Study of Dendritic Cell Vaccine Loaded With Circular RNA Encoding Cryptic Peptide for Patients With HER2-negative Advanced Breast Cancer	NOT_YET_RECRUITING	Breast Neoplasms	[119]
NCT06617585	The Role of CircDENND4C in Epithelial Ovarian Cancer	NOT_YET_RECRUITING	Ovarian Cancer	[120]
NCT06649253	Childhood B-acute Lymphoblastic Leukemia and Role of CD9 Gene Regulation in Relapse	RECRUITING	Leukemia, Lymphoblastic, Acute, Pediatric	[121, 122]
NCT04464122	Rediscovering Biomarkers for the Diagnosis and Early Treatment Response in NEN (REBORN)	RECRUITING	Neuroendocrine Tumors|Neuroendocrine Neoplasm|Neuroendocrine Tumor Grade 1|Neuroendocrine Tumor Grade 2|Neuroendocrine Carcinoma	[122]
NCT07081984	Study of TI-0093 Injection With Recurrent/Metastatic HPV-16 Positive Solid Tumors	NOT_YET_RECRUITING	HPV 16 + Recurrent or Metastatic Cancer|HNSCC|Cervical Cancer	[123]
NCT06717295	The CCANED-CIPHER Study: Early Cancer Detection and Treatment Response Monitoring Using AI-Based Platelet and Immune Cell Transcriptomic Profiling	NOT_YET_RECRUITING	Brest Cancer|Lung Cancer (NSCLC)|Pancreatic Cancer, Adult|Prostate Cancers|Ovarian Cancer|Colorectal Cancer|Glioblastoma (GBM)|Liver Carcinoma	[124]
NCT03334708	A Study of Blood-Based Biomarkers for Pancreas Adenocarcinoma	RECRUITING	Pancreatic Cancer|Pancreatic Diseases|Pancreatitis|Pancreatic Cyst	[125]

## Supplementary Information

Below is the link to the electronic supplementary material.

Supplementary material 1 (pdf 418 KB)

## Data Availability

No datasets were generated or analysed during the current study.

## References

[1] Pamudurti NR, Bartok O, Jens M et al (2017) Translation of CircRNAs. Mol Cell 66(1):9 28344080 10.1016/j.molcel.2017.02.021 PMC5387669

[2] Li W, Liu JQ, Chen M et al (2022) Circular RNA in cancer development and immune regulation. J Cell Mol Med 26(6):1785–1798 33277969 10.1111/jcmm.16102 PMC8918416

[3] Chen LL (2016) The biogenesis and emerging roles of circular RNAs. Nat Rev Mol Cell Biol 17(4):205–211 26908011 10.1038/nrm.2015.32

[4] Chen LL, Yang L (2015) Regulation of circRNA biogenesis. RNA Biol 12(4):381–388 25746834 10.1080/15476286.2015.1020271 PMC4615371

[5] Kristensen LS, Andersen MS, Stagsted LVW et al (2019) The biogenesis, biology and characterization of circular RNAs. Nat Rev Genet 20(11):675–691 31395983 10.1038/s41576-019-0158-7

[6] He AT, Liu J, Li F et al (2021) Targeting circular RNAs as a therapeutic approach: current strategies and challenges. Signal Transduct Target Ther 6(1):185 34016945 10.1038/s41392-021-00569-5 PMC8137869

[7] Li M, Wang Y, Wu P, Zhang S, Gong Z, Liao Q, Xiong W (2023) Application prospect of circular RNA-based neoantigen vaccine in tumor immunotherapy. Cancer lett 563:216190 37062328 10.1016/j.canlet.2023.216190

[8] Kaviyarasan V, Das A, Deka D et al (2024) Advancements in immunotherapy for colorectal cancer treatment: a comprehensive review of strategies, challenges, and future prospective. Int J Colorectal Dis. 10.1007/s00384-024-04790-w 39731596 10.1007/s00384-024-04790-w PMC11682016

[9] Xiong ZJ, Sun LL, Yang H et al (2023) Ni-alginate hydrogel microspheres with sustained interleukin 2 release to boost cytokine-based cancer immunotherapy. Adv Funct Mater. 10.1002/adfm.202211423

[10] Salzman J, Chen RE, Olsen MN et al (2013) Cell-type specific features of circular RNA expression. PLoS Genet 9(9):e1003777 24039610 10.1371/journal.pgen.1003777 PMC3764148

[11] Xia S, Feng J, Lei L, Hu J, Xia L, Wang J, He C (2017) Comprehensive characterization of tissue-specific circular RNAs in the human and mouse genomes. Brief bioinform 18(6):984–992 27543790 10.1093/bib/bbw081

[12] Chen Z, He L, Zhao L et al (2022) CircREEP3 drives colorectal cancer progression via activation of FKBP10 transcription and restriction of antitumor immunity. Adv Sci 9(13):e2105160 10.1002/advs.202105160 PMC9069384 35233964

[13] Song J, Zheng J, Liu X et al (2022) A novel protein encoded by ZCRB1-induced circHEATR5B suppresses aerobic glycolysis of GBM through phosphorylation of JMJD5. J Exp Clin Cancer Res 41(1):171 35538499 10.1186/s13046-022-02374-6 PMC9086421

[14] Gao J, Ao YQ, Zhang LX et al (2022) Exosomal circZNF451 restrains anti-PD1 treatment in lung adenocarcinoma via polarizing macrophages by complexing with TRIM56 and FXR1. J Exp Clin Cancer Res 41(1):295 36209117 10.1186/s13046-022-02505-z PMC9547453

[15] Zheng R, Zhang K, Tan S, Gao F, Zhang Y, Xu W, Wang M (2022) Exosomal circLPAR1 functions in colorectal cancer diagnosis and tumorigenesis through suppressing BRD4 via METTL3–eIF3h interaction. Mol cancer 21(1):49 35164758 10.1186/s12943-021-01471-y PMC8842935

[16] Zhang X, Xu YH, Ma LF et al (2022) Essential roles of exosome and circRNA_101093 on ferroptosis desensitization in lung adenocarcinoma. Cancer Commun 42(4):287–313 10.1002/cac2.12275 PMC9017758 35184419

[17] Chen C, Liu Y, Liu L et al (2023) Exosomal circTUBGCP4 promotes vascular endothelial cell tipping and colorectal cancer metastasis by activating Akt signaling pathway. J Exp Clin Cancer Res 42(1):46 36793126 10.1186/s13046-023-02619-y PMC9930311

[18] Wang C, Liu Y, Zuo Z et al (2024) Dual role of exosomal circCMTM3 derived from GSCs in impeding degradation and promoting phosphorylation of STAT5A to facilitate vasculogenic mimicry formation in glioblastoma. Theranostics 14(14):5698–5724 39310105 10.7150/thno.97057 PMC11413784

[19] Wang D, Wang S, Jin M et al (2024) Hypoxic exosomal circPLEKHM1-mediated crosstalk between tumor cells and macrophages drives lung cancer metastasis. Adv Sci 11(22):e2309857 10.1002/advs.202309857 PMC11165461 38509870

[20] Sun Z, Xu Y, Shao B et al (2024) Exosomal circPOLQ promotes macrophage M2 polarization via activating IL-10/STAT3 axis in a colorectal cancer model. J Immunother Cancer. 10.1136/jitc-2023-008491 38782541 10.1136/jitc-2023-008491 PMC11116870

[21] Chen Q, Wang H, Li Z et al (2022) Circular RNA ACTN4 promotes intrahepatic cholangiocarcinoma progression by recruiting YBX1 to initiate FZD7 transcription. J Hepatol 76(1):135–147 34509526 10.1016/j.jhep.2021.08.027

[22] Chen RX, Liu SC, Kan XC et al (2024) CircUGP2 suppresses intrahepatic cholangiocarcinoma progression via p53 signaling through interacting with PURB to regulate ADGRB1 transcription and sponging miR-3191-5p. Adv Sci 11(38):e2402329 10.1002/advs.202402329 PMC11481218 39120980

[23] Fei D, Wang F, Wang Y et al (2024) Circular RNA ACVR2A promotes the progression of hepatocellular carcinoma through mir-511-5p targeting PI3K-Akt signaling pathway. Mol Cancer 23(1):159 39107843 10.1186/s12943-024-02074-z PMC11302160

[24] Ding L, Wang R, Zheng Q et al (2022) CircPDE5A regulates prostate cancer metastasis via controlling WTAP-dependent N6-methyladenisine methylation of EIF3C mRNA. J Exp Clin Cancer Res 41(1):187 35650605 10.1186/s13046-022-02391-5 PMC9161465

[25] Du J, Lan T, Liao H et al (2022) CircNFIB inhibits tumor growth and metastasis through suppressing MEK1/ERK signaling in intrahepatic cholangiocarcinoma. Mol Cancer 21(1):18 35039066 10.1186/s12943-021-01482-9 PMC8762882

[26] Li X, Wang J, Lin W et al (2022) circEXOC6B interacting with RRAGB, an mTORC1 activator, inhibits the progression of colorectal cancer by antagonizing the HIF1A-RRAGB-mTORC1 positive feedback loop. Mol Cancer 21(1):135 35739524 10.1186/s12943-022-01600-1 PMC9219196

[27] Pan Z, Zhao R, Li B et al (2022) EWSR1-induced circNEIL3 promotes glioma progression and exosome-mediated macrophage immunosuppressive polarization via stabilizing IGF2BP3. Mol Cancer 21(1):16 35031058 10.1186/s12943-021-01485-6 PMC8759291

[28] Yang R, Chen H, Xing L et al (2022) Hypoxia-induced circWSB1 promotes breast cancer progression through destabilizing p53 by interacting with USP10. Mol Cancer 21(1):88 35351136 10.1186/s12943-022-01567-z PMC8961958

[29] Xu Z, Chen S, Liu R et al (2022) Circular RNA circPOLR2A promotes clear cell renal cell carcinoma progression by facilitating the UBE3C-induced ubiquitination of PEBP1 and thereby, activating the ERK signaling pathway. Mol Cancer 21(1):146 35840930 10.1186/s12943-022-01607-8 PMC9284792

[30] Ling Y, Liang G, Lin Q et al (2022) CircCDYL2 promotes trastuzumab resistance via sustaining HER2 downstream signaling in breast cancer. Mol Cancer 21(1):8 34980129 10.1186/s12943-021-01476-7 PMC8722291

[31] Rossi F, Beltran M, Damizia M, Grelloni C, Colantoni A, Setti A, Bozzoni I (2022) Circular RNA ZNF609/CKAP5 mRNA interaction regulates microtubule dynamics and tumorigenicity. Mol cell 82(1):75–89 34942120 10.1016/j.molcel.2021.11.032 PMC8751636

[32] Li Y, Wang Z, Yang J et al (2024) CircTRIM1 encodes TRIM1-269aa to promote chemoresistance and metastasis of TNBC via enhancing CaM-dependent MARCKS translocation and PI3K/AKT/mTOR activation. Mol Cancer 23(1):102 38755678 10.1186/s12943-024-02019-6 PMC11097450

[33] Xiong L, Liu HS, Zhou C et al (2023) A novel protein encoded by circINSIG1 reprograms cholesterol metabolism by promoting the ubiquitin-dependent degradation of INSIG1 in colorectal cancer. Mol Cancer 22(1):72 37087475 10.1186/s12943-023-01773-3 PMC10122405

[34] Yang Y, Fan X, Mao M et al (2017) Extensive translation of circular RNAs driven by N(6)-methyladenosine. Cell Res 27(5):626–641 28281539 10.1038/cr.2017.31 PMC5520850

[35] Wu N, Yuan Z, Du KY et al (2019) Translation of yes-associated protein (YAP) was antagonized by its circular RNA via suppressing the assembly of the translation initiation machinery. Cell Death Differ 26(12):2758–2773 31092884 10.1038/s41418-019-0337-2 PMC7224378

[36] Li Z, Huang C, Bao C, Chen L, Lin M, Wang X, Shan G (2015) Exon-intron circular RNAs regulate transcription in the nucleus. Nat struct mol biol 22(3):256–264 25664725 10.1038/nsmb.2959

[37] Hanahan D, Weinberg RA (2011) Hallmarks of cancer: the next generation. Cell 144(5):646–674 21376230 10.1016/j.cell.2011.02.013

[38] Conn VM, Gabryelska M, Toubia J, Kirk K, Gantley L, Powell JA, Conn SJ (2023) Circular RNAs drive oncogenic chromosomal translocations within the MLL recombinome in leukemia. Cancer Cell 41(7):1309–1326 37295428 10.1016/j.ccell.2023.05.002

[39] Wang Z, Li Y, Yang J, Sun Y, He Y, Wang Y, Yang Q (2024) CircCFL1 Promotes TNBC Stemness and Immunoescape via Deacetylation-Mediated c-Myc Deubiquitylation to Facilitate Mutant TP53 Transcription. Adv Sci 11(34):2404628 10.1002/advs.202404628 PMC11425638 38981022

[40] Liu Y, Fang S, Lin T et al (2024) Circular RNA circNIPBL regulates TP53-H179R mutations in NNK-induced bronchial epithelial carcinogenesis. Environ Int 190:108829 38908277 10.1016/j.envint.2024.108829

[41] Hedayati N, Babaei Aghdam Z, Rezaee M et al (2025) Recent insights into the angioregulatory role of long non-coding RNAs and circular RNAs in gliomas: from signaling pathways to clinical aspects. Curr Med Chem 32(16):3169–3192 38258785 10.2174/0109298673259378231031061149

[42] Hao X, Zhang Y, Shi X et al (2022) Circpak1 promotes the progression of hepatocellular carcinoma via modulation of YAP nucleus localization by interacting with 14-3-3ζ. J Exp Clin Cancer Res 41(1):281 36131287 10.1186/s13046-022-02494-z PMC9494907

[43] Yu L, Zhu H, Wang Z et al (2022) Circular RNA circFIRRE drives osteosarcoma progression and metastasis through tumorigenic-angiogenic coupling. Mol Cancer 21(1):167 35986280 10.1186/s12943-022-01624-7 PMC9389772

[44] Mao Y, Wang J, Wang Y et al (2024) Hypoxia induced exosomal circ-ZNF609 promotes pre-metastatic niche formation and cancer progression via miR-150-5p/VEGFA and HuR/ZO-1 axes in esophageal squamous cell carcinoma. Cell Death Discov 10(1):133 38472174 10.1038/s41420-024-01905-8 PMC10933275

[45] Yu W, Chen D, Ma L et al (2025) EIF4A3-induced circ_0059914 promoted angiogenesis and EMT of glioma via the miR-1249/VEGFA pathway. Mol Neurobiol 62(1):973–987 38951469 10.1007/s12035-024-04319-w

[46] Li X, Wang C, Zhang H et al (2023) Circfndc3b accelerates vasculature formation and metastasis in oral squamous cell carcinoma. Cancer Res 83(9):1459–1475 36811957 10.1158/0008-5472.CAN-22-2585 PMC10152237

[47] Schito L, Semenza GL (2016) Hypoxia-inducible factors: master regulators of cancer progression. Trends Cancer 2(12):758–770 28741521 10.1016/j.trecan.2016.10.016

[48] Lu W, Li L, Li L et al (2024) Circular RNA circ_0101675 promotes NSCLC cell proliferation, migration, invasion, angiogenesis and immune evasion by sponging miR-607/PDL1 axis. Biochem Genet 62(3):1539–1555 37646893 10.1007/s10528-023-10493-8

[49] Xi Y, Shen Y, Wu D et al (2022) Circbcar3 accelerates esophageal cancer tumorigenesis and metastasis via sponging mir-27a-3p. Mol Cancer 21(1):145 35840974 10.1186/s12943-022-01615-8 PMC9284725

[50] Peuhu E, Virtakoivu R, Mai A et al (2017) Epithelial vimentin plays a functional role in mammary gland development. Development 144(22):4103–4113 28947532 10.1242/dev.154229

[51] Zhang Y, Liu Z, Zhong Z et al (2024) A tumor suppressor protein encoded by circKEAP1 inhibits osteosarcoma cell stemness and metastasis by promoting vimentin proteasome degradation and activating anti-tumor immunity. J Exp Clin Cancer Res 43(1):52 38383479 10.1186/s13046-024-02971-7 PMC10880370

[52] Rong Z, Xu J, Yang J et al (2024) Circrreb1 mediates metabolic reprogramming and stemness maintenance to facilitate pancreatic ductal adenocarcinoma progression. Cancer Res. 10.1158/0008-5472.CAN-23-3596 39288082 10.1158/0008-5472.CAN-23-3596

[53] Zheng S, Tian Q, Yuan Y et al (2023) Extracellular vesicle-packaged circBIRC6 from cancer-associated fibroblasts induce platinum resistance via SUMOylation modulation in pancreatic cancer. J Exp Clin Cancer Res 42(1):324 38012734 10.1186/s13046-023-02854-3 PMC10683239

[54] Jiang X, Guo S, Wang S et al (2022) EIF4A3-induced circARHGAP29 promotes aerobic glycolysis in Docetaxel-resistant prostate cancer through IGF2BP2/c-Myc/LDHA signaling. Cancer Res 82(5):831–845 34965937 10.1158/0008-5472.CAN-21-2988

[55] Li H, Luo F, Jiang X et al (2022) CircITGB6 promotes ovarian cancer cisplatin resistance by resetting tumor-associated macrophage polarization toward the M2 phenotype. J Immunother Cancer. 10.1136/jitc-2021-004029 35277458 10.1136/jitc-2021-004029 PMC8919471

[56] Pan Z, Zheng J, Zhang J et al (2022) A novel protein encoded by exosomal CircATG4B induces oxaliplatin resistance in colorectal cancer by promoting autophagy. Adv Sci 9(35):e2204513 10.1002/advs.202204513 PMC9762280 36285810

[57] Wang X, Chen T, Li C et al (2022) CircRNA-CREIT inhibits stress granule assembly and overcomes doxorubicin resistance in TNBC by destabilizing PKR. J Hematol Oncol 15(1):122 36038948 10.1186/s13045-022-01345-w PMC9425971

[58] Lorentzen CL, Haanen JB, Met Ö, Svane IM (2022) Clinical advances and ongoing trials of mRNA vaccines for cancer treatment. Lancet Oncol 23(10):e450–e458 36174631 10.1016/S1470-2045(22)00372-2 PMC9512276

[59] Ribas A, Wolchok JD (2018) Cancer immunotherapy using checkpoint blockade. Science 359(6382):1350 29567705 10.1126/science.aar4060 PMC7391259

[60] Pan K, Farrukh H, Chittepu V et al (2022) CAR race to cancer immunotherapy: from CAR T, CAR NK to CAR macrophage therapy. J Exp Clin Cancer Res 41(1):119 35361234 10.1186/s13046-022-02327-z PMC8969382

[61] Xiao Y, Yu D (2021) Tumor microenvironment as a therapeutic target in cancer. Pharmacol ther 221:107753 33259885 10.1016/j.pharmthera.2020.107753 PMC8084948

[62] Hu Z, Chen G, Zhao Y et al (2023) Exosome-derived circCCAR1 promotes CD8 + T-cell dysfunction and anti-PD1 resistance in hepatocellular carcinoma. Mol Cancer 22(1):55 36932387 10.1186/s12943-023-01759-1 PMC10024440

[63] Wang B, Liu W, Zhang M et al (2024) Circ_0001947 encapsulated by small extracellular vesicles promotes gastric cancer progression and anti-PD-1 resistance by modulating CD8(+) T cell exhaustion. J Nanobiotechnology 22(1):563 39272146 10.1186/s12951-024-02826-5 PMC11401313

[64] Zhang LX, Gao J, Long X et al (2022) The circular RNA circHMGB2 drives immunosuppression and anti-PD-1 resistance in lung adenocarcinomas and squamous cell carcinomas via the miR-181a-5p/CARM1 axis. Mol Cancer 21(1):110 35525959 10.1186/s12943-022-01586-w PMC9077876

[65] Liu Z, Zheng N, Li J, Li C, Zheng D, Jiang X, Gao X (2022) N6-methyladenosine-modified circular RNA QSOX1 promotes colorectal cancer resistance to anti-CTLA-4 therapy through induction of intratumoral regulatory T cells. Drug Resist Updat 65:100886 36370665 10.1016/j.drup.2022.100886

[66] Zang X, He XY, Xiao CM et al (2024) Circular RNA-encoded oncogenic PIAS1 variant blocks immunogenic ferroptosis by modulating the balance between SUMOylation and phosphorylation of STAT1. Mol Cancer 23(1):207 39334380 10.1186/s12943-024-02124-6 PMC11438063

[67] Propper DJ, Balkwill FR (2022) Harnessing cytokines and chemokines for cancer therapy. Nat Rev Clin Oncol 19(4):237–253 34997230 10.1038/s41571-021-00588-9

[68] Waldmann TA (2018) Cytokines in cancer immunotherapy. Cold Spring Harb Perspect Biol. 10.1101/cshperspect.a028472 29101107 10.1101/cshperspect.a028472 PMC6280701

[69] Yi M, Li T, Niu M et al (2024) Targeting cytokine and chemokine signaling pathways for cancer therapy. Signal Transduct Target Ther 9(1):176 39034318 10.1038/s41392-024-01868-3 PMC11275440

[70] Taniguchi K, Karin M (2014) IL-6 and related cytokines as the critical lynchpins between inflammation and cancer. Semin Immunol 26(1):54–74 24552665 10.1016/j.smim.2014.01.001

[71] Zhao Y, Jia Y, Wang J et al (2024) CircNOX4 activates an inflammatory fibroblast niche to promote tumor growth and metastasis in NSCLC via FAP/IL-6 axis. Mol Cancer 23(1):47 38459511 10.1186/s12943-024-01957-5 PMC10921747

[72] Shi X, Yang J, Liu M, Zhang Y, Zhou Z, Luo W, Li M (2022) Circular RNA ANAPC7 inhibits tumor growth and muscle wasting via PHLPP2–AKT–TGF-β signaling axis in pancreatic cancer. Gastroenterology 162(7):2004–2017 35176309 10.1053/j.gastro.2022.02.017 PMC10428768

[73] Piras R, Ko EY, Barrett C et al (2022) circCsnk1g3- and circAnkib1-regulated interferon responses in sarcoma promote tumorigenesis by shaping the immune microenvironment. Nat Commun 13(1):7243 36433954 10.1038/s41467-022-34872-8 PMC9700836

[74] Li K, Guo J, Ming Y, Chen S, Zhang T, Ma H, Peng Y (2023) A circular RNA activated by TGFβ promotes tumor metastasis through enhancing IGF2BP3-mediated PDPN mRNA stability. Nat Commun 14(1):6876 37898647 10.1038/s41467-023-42571-1 PMC10613289

[75] Bhat AA, Nisar S, Singh M et al (2022) Cytokine- and chemokine-induced inflammatory colorectal tumor microenvironment: emerging avenue for targeted therapy. Cancer Commun 42(8):689–715 10.1002/cac2.12295 PMC9395317 35791509

[76] Li H, Lan T, Liu H et al (2022) IL-6-induced cGGNBP2 encodes a protein to promote cell growth and metastasis in intrahepatic cholangiocarcinoma. Hepatology 75(6):1402–1419 34758510 10.1002/hep.32232 PMC9306806

[77] Peng M, Mo Y, Wang Y et al (2019) Neoantigen vaccine: an emerging tumor immunotherapy. Mol Cancer 18(1):128 31443694 10.1186/s12943-019-1055-6 PMC6708248

[78] Wang F, Cai G, Wang Y et al (2024) Circular RNA-based neoantigen vaccine for hepatocellular carcinoma immunotherapy. MedComm 5(8):e667 39081513 10.1002/mco2.667 PMC11286538

[79] Ren Y, Manoharan T, Liu B et al (2024) Circular RNA as a source of neoantigens for cancer vaccines. J Immunother Cancer. 10.1136/jitc-2023-008402 38508656 10.1136/jitc-2023-008402 PMC10952939

[80] Maalej KM, Merhi M, Inchakalody VP et al (2023) CAR-cell therapy in the era of solid tumor treatment: current challenges and emerging therapeutic advances. Mol Cancer 22(1):20 36717905 10.1186/s12943-023-01723-z PMC9885707

[81] Rafiq S, Hackett CS, Brentjens RJ (2020) Engineering strategies to overcome the current roadblocks in CAR T cell therapy. Nat Rev Clin Oncol 17(3):147–167 31848460 10.1038/s41571-019-0297-y PMC7223338

[82] Wu J, Wu W, Zhou B et al (2024) Chimeric antigen receptor therapy meets mRNA technology. Trends Biotechnol 42(2):228–240 37741706 10.1016/j.tibtech.2023.08.005

[83] Liu C, Shi Q, Huang X et al (2023) mRNA-based cancer therapeutics. Nat Rev Cancer 23(8):526–543 37311817 10.1038/s41568-023-00586-2

[84] Metkar M, Pepin CS, Moore MJ (2024) Tailor made: the art of therapeutic mRNA design. Nat Rev Drug Discov 23(1):67–83 38030688 10.1038/s41573-023-00827-x

[85] Zhang Z, Ma B, Li B et al(2024) Cardiolipin-mimic lipid nanoparticles without antibody modification delivered senolytic in-vivo CAR-T therapy for inflamm-aging*.* bioRxiv 2024.11.21.624667. 10.1016/j.xcrm.2025.102209 PMC12281384 40602406

[86] Uruga H, Mino-Kenudson M (2021) Predictive biomarkers for response to immune checkpoint inhibitors in lung cancer: PD-L1 and beyond. Virchows Arch 478(1):31–44 33486574 10.1007/s00428-021-03030-8

[87] Sholl LM (2022) Biomarkers of response to checkpoint inhibitors beyond PD-L1 in lung cancer. Mod Pathol 35:66–74 34608245 10.1038/s41379-021-00932-5

[88] Rybak-Wolf A, Stottmeister C, Glažar P, Jens M, Pino N, Giusti S, Rajewsky N (2015) Circular RNAs in the Mammalian Brain Are Highly Abundant, Conserved, and Dynamically Expressed. Mol cell 58(5):870–885 25921068 10.1016/j.molcel.2015.03.027

[89] Salzman J, Gawad C, Wang PL et al (2012) Circular rnas are the predominant transcript isoform from hundreds of human genes in diverse cell types. PLoS ONE 7(2):e30733 22319583 10.1371/journal.pone.0030733 PMC3270023

[90] Roy S, Kanda M, Nomura S et al (2022) Diagnostic efficacy of circular RNAs as noninvasive, liquid biopsy biomarkers for early detection of gastric cancer. Mol Cancer 21(1):42 35139874 10.1186/s12943-022-01527-7 PMC8826675

[91] Hansen EB, Fredsøe J, Okholm TLH et al (2022) The transcriptional landscape and biomarker potential of circular RNAs in prostate cancer. Genome Med 14(1):8 35078526 10.1186/s13073-021-01009-3 PMC8788096

[92] Wen N, Peng D, Xiong X, Liu G, Nie G, Wang Y, Cheng N (2024) Cholangiocarcinoma combined with biliary obstruction: an exosomal circRNA signature for diagnosis and early recurrence monitoring. Signal transduct target ther 9(1):107 38697972 10.1038/s41392-024-01814-3 PMC11636852

[93] Xu C, Jun E, Okugawa Y et al (2024) A circulating panel of circRNA biomarkers for the noninvasive and early detection of pancreatic ductal adenocarcinoma. Gastroenterology 166(1):178-190.e16 37839499 10.1053/j.gastro.2023.09.050 PMC10843014

[94] Lin J, Wang X, Zhai S et al (2022) Hypoxia-induced exosomal circPDK1 promotes pancreatic cancer glycolysis via c-myc activation by modulating miR-628-3p/BPTF axis and degrading BIN1. J Hematol Oncol 15(1):128 36068586 10.1186/s13045-022-01348-7 PMC9450374

[95] Zheng J, Yan X, Lu T et al (2023) CircFOXK2 promotes hepatocellular carcinoma progression and leads to a poor clinical prognosis via regulating the Warburg effect. J Exp Clin Cancer Res 42(1):63 36922872 10.1186/s13046-023-02624-1 PMC10018916

[96] Miao J, Wang B, Shao R et al (2021) CircUSP36 knockdown alleviates oxidized low-density lipoprotein-induced cell injury and inflammatory responses in human umbilical vein endothelial cells via the miR-20a-5p/ROCK2 axis. Int J Mol Med 47(4):40 33576448 10.3892/ijmm.2021.4873 PMC7891832

[97] Huang JG, Tang X, Wang JJ et al (2021) A circular RNA, circUSP36, accelerates endothelial cell dysfunction in atherosclerosis by adsorbing miR-637 to enhance WNT4 expression. Bioengineered 12(1):6759–6770 34519627 10.1080/21655979.2021.1964891 PMC8806706

[98] Wan H, You T, Luo W (2021) Circ_0003204 regulates cell growth, oxidative stress, and inflammation in ox-LDL-induced vascular endothelial cells via regulating miR-942-5p/HDAC9 axis. Front Cardiovasc Med 8:646832 33869307 10.3389/fcvm.2021.646832 PMC8047481

[99] Zhang D, Zhang G, Yu K et al (2022) Circ_0003204 knockdown protects endothelial cells against oxidized low-density lipoprotein-induced injuries by targeting the miR-491-5p-ICAM1 pathway. J Thromb Thrombolysis 53(2):302–312 34797473 10.1007/s11239-021-02606-0

[100] Zhao Q, Lu YH, Wang X, Zhang XJ (2020) Circ_USP36/miR-182-5p/KLF5 axis regulates the ox-LDL-induced injury in human umbilical vein smooth muscle cells. Am j transl res 12(12):7855 33437365 PMC7791491

[101] Ji N, Wang Y, Gong X et al (2021) CircMTO1 inhibits ox-LDL-stimulated vascular smooth muscle cell proliferation and migration via regulating the miR-182-5p/RASA1 axis. Mol Med 27(1):73 34238206 10.1186/s10020-021-00330-2 PMC8268171

[102] Su G, Sun G, Lv J et al (2021) Hsa_circ_0004831 downregulation is partially responsible for atorvastatinalleviated human umbilical vein endothelial cell injuries induced by ox-LDL through targeting the miR-182-5p/CXCL12 axis. BMC Cardiovasc Disord 21(1):221 33932991 10.1186/s12872-021-01998-4 PMC8088699

[103] Zhang P, Wang W, Li M (2022) Role and mechanism of circular RNA circ_0050486 in regulating oxidized low-density lipoprotein-induced injury in endothelial cells. Clin Hemorheol Microcirc 82(2):107–124 35723090 10.3233/CH-211259

[104] Chen J, Hei R, Chen C, Wu X, Han T, Bian H, Zheng Q (2023) CircCRIM1 suppresses osteosarcoma progression via sponging miR146a-5p and targeting NUMB. Am J Cancer Res 13(8):3463 37693139 PMC10492126

[105] Du Y, Liu X, Zhang S et al (2021) Circcrim1 promotes ovarian cancer progression by working as cernas of CRIM1 and targeting miR-383-5p/ZEB2 axis. Reprod Biol Endocrinol 19(1):176 34847936 10.1186/s12958-021-00857-3 PMC8630901

[106] He W, Zhou X, Mao Y, Wu Y, Tang X, Yan S, Tang S (2022) CircCRIM1 promotes nasopharyngeal carcinoma progression via the miR-34c-5p/FOSL1 axis. Eur j med res 27(1):59 35484574 10.1186/s40001-022-00667-2 PMC9052594

[107] Li Y, Jiang B, Zeng L, Tang Y, Qi X, Wan Z, Wu Y (2023) Adipocyte-derived exosomes promote the progression of triple-negative breast cancer through circCRIM1-dependent OGA activation. Environ Res 239:117266 37775001 10.1016/j.envres.2023.117266

[108] Triska J, Mathew C, Zhao Y et al (2023) Circular RNA as therapeutic targets in atherosclerosis: are we running in circles? J Clin Med. 10.3390/jcm12134446 37445481 10.3390/jcm12134446 PMC10342353

[109] Du A, Li S, Zhou Y, Disoma C, Liao Y, Zhang Y, Xia Z (2022) M6A-mediated upregulation of circMDK promotes tumorigenesis and acts as a nanotherapeutic target in hepatocellular carcinoma. Mol cancer 21(1):109 35524319 10.1186/s12943-022-01575-z PMC9074191

[110] Xu L, Wang P, Li L et al (2023) CircPSD3 is a promising inhibitor of uPA system to inhibit vascular invasion and metastasis in hepatocellular carcinoma. Mol Cancer 22(1):174 37884951 10.1186/s12943-023-01882-z PMC10601121

[111] Wei W, Liu K, Huang X et al (2024) EIF4A3-mediated biogenesis of circSTX6 promotes bladder cancer metastasis and cisplatin resistance. J Exp Clin Cancer Res 43(1):2 38163881 10.1186/s13046-023-02932-6 PMC10759346

[112] Ma Q, Yang F, Huang B et al (2022) Circarid1a binds to IGF2BP3 in gastric cancer and promotes cancer proliferation by forming a circARID1A-IGF2BP3-SLC7A5 RNA-protein ternary complex. J Exp Clin Cancer Res 41(1):251 35986300 10.1186/s13046-022-02466-3 PMC9389715

[113] Xu L, Ma X, Zhang X et al (2023) Hsa_circ_0007919 induces LIG1 transcription by binding to FOXA1/TET1 to enhance the DNA damage response and promote gemcitabine resistance in pancreatic ductal adenocarcinoma. Mol Cancer 22(1):195 38044421 10.1186/s12943-023-01887-8 PMC10694898

[114] Yang M, Hu H, Wu S et al (2022) EIF4A3-regulated circ_0087429 can reverse EMT and inhibit the progression of cervical cancer via miR-5003-3p-dependent upregulation of OGN expression. J Exp Clin Cancer Res 41(1):165 35513835 10.1186/s13046-022-02368-4 PMC9069757

[115] CIRcular and Non-coding RNAs as Clinically USeful Biomarkers in Pancreaticobiliary Cancers. 2020.

[116] Deciphering the Role of Circular RNAs in the Pathogenesis and Therapy Resistance of ALKpositive Anaplastic Large-cell Lymphoma. F. Institut National de la Santé Et de la Recherche Médicale, Editor. 2023.

[117] The Value of circRNAs (hsa_circ_0004001) in Early Diagnosis of HCC. 2023.

[118] Circular RNA and Chemerin in Blood of Breast Cancer Patients. 2023.

[119] A Single Arm Clinical Study of Dendritic Cell Vaccine Loaded With Circular RNA Encoding Cryptic Peptide for Patients With HER2-negative Advanced Breast Cancer. 2024.

[120] The Role of CircDENND4C in Epithelial Ovarian Cancer. 2023.

[121] REALL CD9 : Molecular Mechanisms Involved in Relapses of Childhood B-acute Lymphoblastic Leukaemia, Role of Non-coding RNA in CD9 Gene Regulation. 2024.

[122] Rediscovering Biomarkers for the Diagnosis and Early Treatment Response in NEN. REBORN Study. 2020.

[123] A Phase I Clinical Study to Evaluate the Safety, Tolerability, Efficacy and Immunogenicity of TI-0093 Injection in Patients With Recurrent/Metastatic HPV-16 Positive Solid Tumors. 2025.

[124] The CCANED-CIPHER Study: Early Cancer Detection and Treatment Response Monitoring Using AI-Based Platelet and Immune Cell Transcriptomic Profiling. 2024.

[125] Development of Biomarkers for the Early Detection, Surveillance and Monitoring of Pancreatic Ductal Adenocarcinoma. C. Sheba Medical, U. Weill Medical College of Cornell, and S. Weizmann Institute of, Editors. 2017.

