# Supplementary material for: Circular RNAs in cancer: Its biogenesis, functions, relationships with cancer progression, applications in immunotherapy and biomarker potentials
Source: Cancer Immunol Immunother. 2026 Mar 31;75(4):125. doi: 10.1007/s00262-025-04176-z (PMC13038836; doi:10.1007/s00262-025-04176-z)
Supplement: Supplementary file 1 — Supplementary material 1 (pdf 418 KB) [file 262_2025_4176_MOESM1_ESM.pdf]

Circular RNAs in Cancer: Its Biogenesis, Functions, Relationships with Cancer Progression, Applications in Immunotherapy and Biomarker Potentials

Yusen Gao[a], Lingling Xu[b], Ruihua Sun[c], Luping Gao[d], Guoliang Wang[e], Jiewen Zhang[c,*], Di Zhu[f,*]

[a] School of Pharmacy, Fudan University

[b] Zhejiang MegaLife Health Technology Group Co., Ltd

[c] Department of Neurology, Henan Provincial People's Hospital, Zhengzhou University People's Hospital, Zhengzhou, Henan, 450003, China

[d] Guangxi Key Laboratory of Tumor Immunology and Microenvironmental Regulation, Guilin Medical University, Guilin, 541004, Chin

[e] UniTTEC Co., Ltd

[f] Department of Pharmacology, School of Basic Medical Sciences, Fudan University

[*] Correspondence to:

Di Zhu, Department of Pharmacology, School of Basic Medical Sciences, Fudan University, Shanghai, Shanghai, 200433, China

Jiewen Zhang, Department of Neurology, Henan Provincial People's Hospital, Zhengzhou University People's Hospital, Zhengzhou, Henan, 450003, China

Email: zhangjiewen9900@126.com

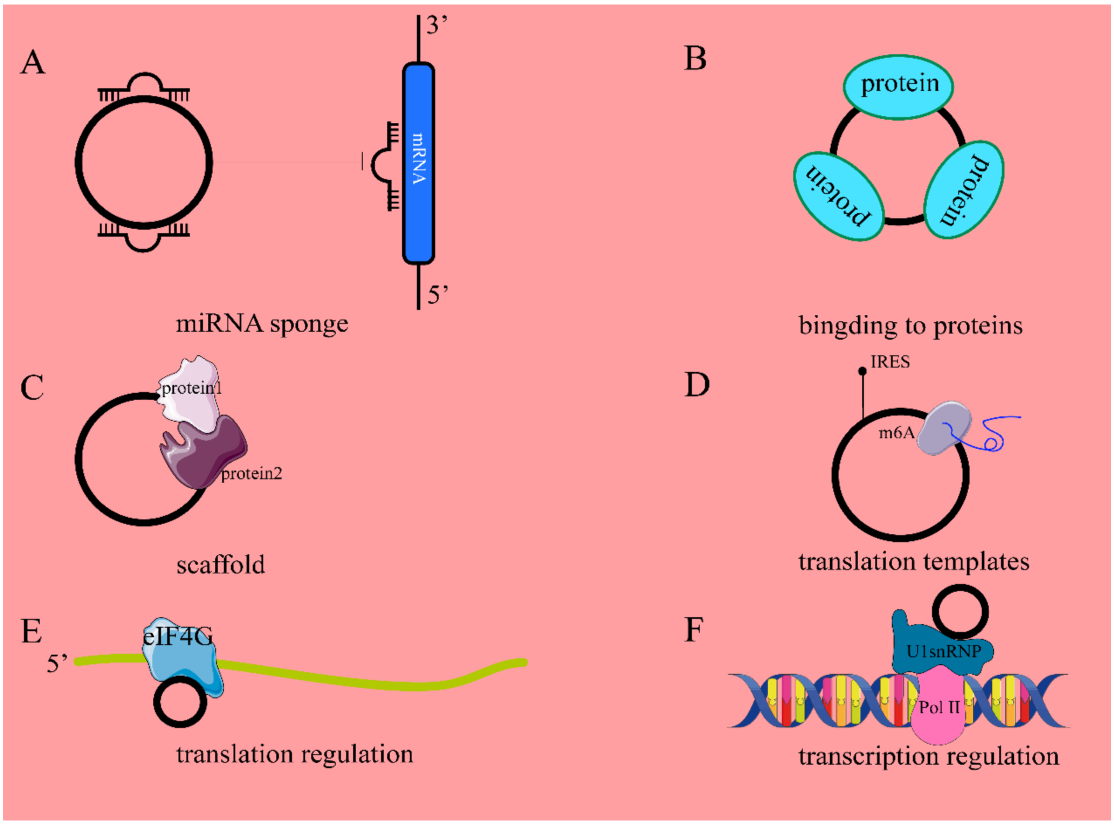

Fig. S1 The functional mechanisms of circRNAs **A.** They act as sponges for microRNAs, thereby reducing their inhibitory effect on target messenger RNAs. **B.** Binding to target proteins to affect their functions. **C.** Serving as a scaffold between proteins or RNA-protein to facilitate mutual interaction. **D.** Encoding proteins, presumably with an internal ribosome entry site (IRES) or m6A modification, which facilitates the initiation of translation. **E.** Regulation of translation by interaction with eukaryotic initiation factors (eIFs), including eIF4G and eIF3h. **F.** CircRNAs may bind factors such as U1 snRNP through RNA-RNA interaction, thereby regulating translation.

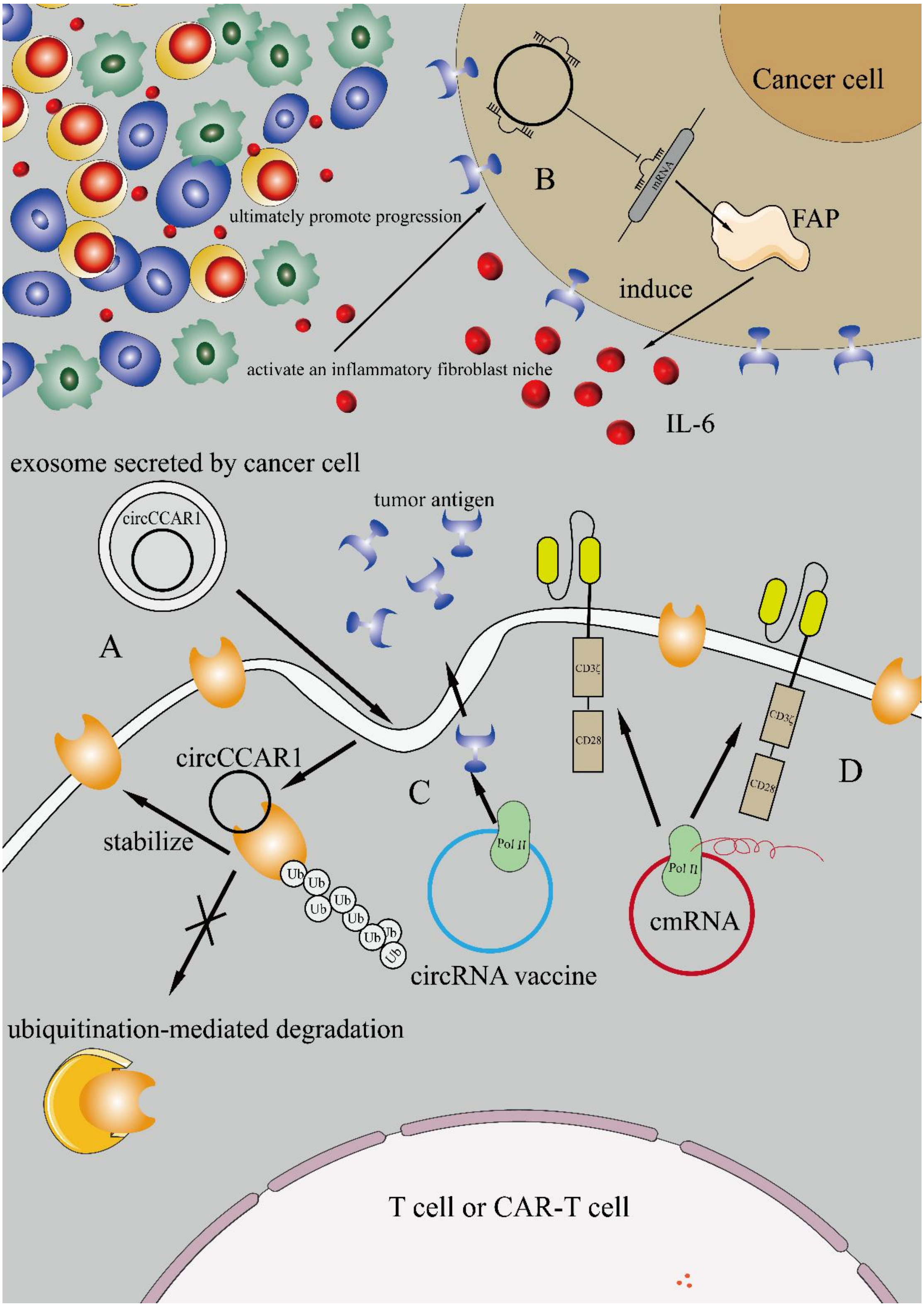

Fig. S2 A. CircRNAs in ICB CircCCAR1 prevents PD-1 from being degraded by ubiquitination by directly binding to PD-1. The binding point of circCCAR1 in PD-1 may be a potential target for blocking. Additionally, circRNAs that are secreted in exosomes are easily detectable in serum and may be exploited as biomarkers. **B. CircRNAs and cytokines:** In the context of non-small cell lung cancer (NSCLC), circNOX4 can upregulate fibroblast activation protein (FAP) by sponging microRNA-329-5p. The circNOX4/miR-329-5p/FAP axis activates an inflammatory fibroblast niche by secreting IL-6, which

ultimately promotes NSCLC progression. Disrupting the circNOX4/IL-6 axis significantly suppresses tumor growth and metastatic colonization. **C&D. Application of circRNAs in cancer vaccines and CAR-T therapies:** Both therapies require an agent to translate CARs or tumor antigens. circRNAs can be used to improve efficiency and effectiveness due to their stability. Additionally, new circRNAs that can translate into neoantigens are possibly produced in some cancer cell subtypes.
